# Fluorine-Mediated Molten-Salt Crystallization Regulates Phase Evolution and Structural Stability in Sodium Layered Oxides

**DOI:** 10.1021/jacs.6c10977

**Published:** 2026-10-21

**Authors:** Chenguang Shi, Junyi Yao, Xiaowen Zhan, Jeongok Choe, Xixian Yang, He Zhou, Amir Ashraf Elnashar, Rupayan Ghosh, Callum Connor, Anika Tabassum Promi, Weibo Huang, Hongyu Wang, Sooyeon Hwang, Kim Kisslinger, Si Athena Chen, Dali Yang, Lu Ma, Hui Zhong, Jianming Bai, Xianghui Xiao, Dennis Nordlund, David Reed, Xiaolin Li, Kejie Zhao, Feng Lin

**Affiliations:** † Department of Chemistry, 1757 Virginia Tech, Blacksburg, Virginia 24061, United States; ‡ School of Engineering, 6752 Brown University, Providence, Rhode Island 02912, United States; § School of Mechanical Engineering, 311308 Purdue University, West Lafayette, Indiana 47907, United States; ∥ Institute for Critical Technology and Applied Science, Virginia Tech, Blacksburg, Virginia 24061, United States; ⊥ Center for Functional Nanomaterials, 8099 Brookhaven National Laboratory, Upton, New York 11973, United States; # Neutron Scattering Division, 6146 Oak Ridge National Laboratory, Oak Ridge, Tennessee 37830, United States; ∇ National Synchrotron Light Source II, Brookhaven National Laboratory, Upton, New York 11973, United States; ○ Stanford Synchrotron Radiation Light Source, 6429 SLAC National Accelerator Laboratory, Menlo Park, California 94025, United States; ◆ Energy and Environmental Directorate, 6865 Pacific Northwest National Laboratory, Richland, Washington 99352, United States

## Abstract

Ni- and Co-free P2-type Fe–Mn layered oxides with high Na concentrations are promising cathodes for sodium-ion batteries, but their practical use is limited by phase instability during Na-excess synthesis and structural degradation during cycling and storage. Although large single-crystal-like architectures could improve stability, controlled phase-selective crystallization in these cathode systems remains challenging. Here, we show that fluorine-assisted molten-salt synthesis enables controlled formation of large-particle Fe–Mn–Ti P2 cathodes with enhanced phase selectivity and structural coherence. Compared with Na-excess processing alone, NaF-assisted flux growth expands the P2 phase stability window and promotes large particles with uniform morphologies while preserving local layered ordering. Multiscale characterization further reveals more-reversible structural evolution and more homogeneous Mn redox behavior in the NaF-assisted material, together with a gradient-like fluorine distribution involving surface-enriched NaF-like species and lower level interior fluorine environments. These coupled structural, morphological, and chemical features collectively mitigate crack formation and deliver good cycling stability. In summary, NaF-assisted molten-salt synthesis shows promise in addressing key limitations of Ni- and Co-free P2-type Fe–Mn layered cathodes.

## Graphical abstract

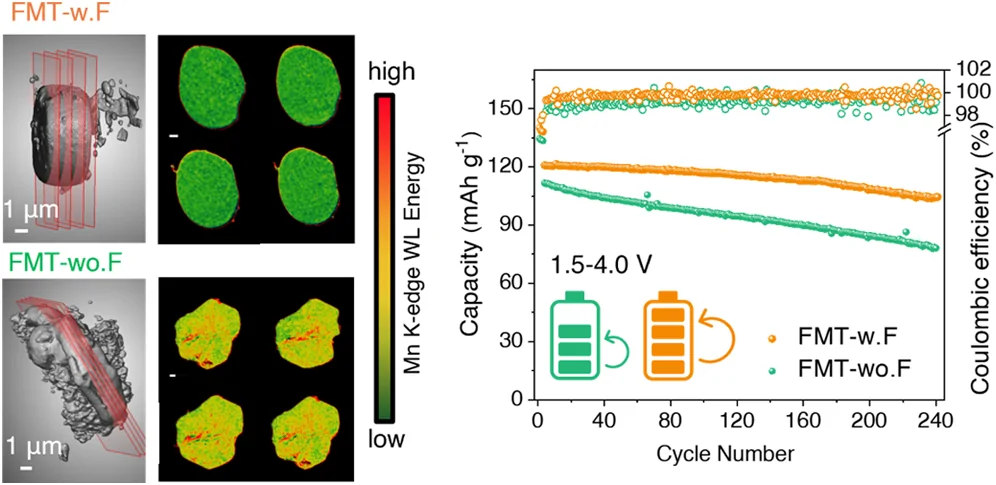

## Introduction

Sodium-ion batteries are emerging as promising alternatives to lithium-ion systems for large-scale energy storage owing to the abundance, low cost, and broad accessibility of sodium resources.^1–3^ Among the various cathode chemistries under development, layered sodium transition-metal (TM) oxides have attracted particular attention because of their high theoretical capacities and structural tunability.^4,5^ Depending on sodium coordination environments and stacking sequences, several layered polymorphs can form, including O3-, P3-, and P2-type phases.^6,7^

Among these motifs, the P2 structure, characterized by prismatic sodium coordination and relatively open diffusion channels, has emerged as one of the most attractive candidates for sodium-ion cathodes.^8^ Compared with O3- and P3-type phases, P2 layered oxides often exhibit balanced sodium mobility, competitive reversible capacity, and comparatively stable lattice evolution under practical operating conditions.^9,10^ In Fe–Mn-based multielement systems composed of earth-abundant elements, P2-type structures therefore represent a compelling pathway toward cost-effective sodium-ion technologies. However, thermodynamic favorability does not guarantee structural robustness in multielement systems. The delicate coupling between sodium chemical potential, TM redox equilibria, and crystallization kinetics governs whether the desired P2 stacking sequence is selectively stabilized.^6,11^ Small perturbations in local composition or sodium activity during synthesis can induce stacking faults, local distortions, and phase impurities.^12^ In addition, the high surface reactivity of Fe–Mn layered oxides often leads to poor tolerance to ambient exposure, further complicating materials processing and storage.^13^ Moreover, as P2 layered oxides often operate through coupled cation–anion redox and anisotropic structural changes, maintaining structural coherence across large particles remains intrinsically challenging.^14,15^ Thus, the central question is not only how to access the P2 phase but also how to stabilize it.

To address the intrinsic structural instability in layered oxides, microstructural engineering has emerged as a central strategy. In lithium layered cathodes, the construction of large single-crystal particles has proven to be highly effective in enhancing mechanical integrity and suppressing intergranular cracking during cycling.^16,17^ By eliminating grain boundaries and reducing defect density, single-crystal architecture promotes more coherent lattice evolution and improved long-term durability. Extending this concept to sodium-layered oxides, however, is not straightforward. Compared with lithium systems, sodium layered materials exhibit more complex polymorphism, larger ionic radius effects, and stronger coupling among Na content, ordering, and stacking sequence. As a result, controlling crystallization pathways and selectively stabilizing a desired motif, such as the P2 structure, while simultaneously promoting large-particle growth, remains challenging.^18,19^ Achieving this requires coordinated regulation of nucleation kinetics, sodium activity, and the local chemical environment during high-temperature synthesis.^6,11^

Beyond microstructural control, compositional engineering provides an additional stabilization pathway. Cation substitution has been widely employed to tune the redox potential and mitigate phase transitions.^20^ However, because cation doping primarily modifies local TM coordination environments,^21^ its influence on global crystallization dynamics and stacking stability can be limited. In contrast, regulating the anion framework may offer an alternative route for structural control. Since the anion sublattice defines the bonding backbone of layered oxides and governs TM-oxygen hybridization, modifying anion chemistry can influence lattice rigidity, redox balance, and phase evolution at a broader scale.^22,23^ Integrating anion-level regulation with large-particle growth therefore presents a mechanistically coherent strategy to stabilize multicomponent sodium layered cathodes.^24^

Here, we demonstrate that coupling Na-rich processing with fluorine-assisted molten-salt synthesis enables controlled crystallization of large-particle Fe–Mn–Ti P2 cathodes. Through a systematic comparison of Na-excess and F-assisted routes, we show that NaF addition expands the phase stability window and regulates crystallization dynamics, leading to phase-selective P2 particles with uniform morphologies. Multiscale characterization reveals moderated TM redox evolution and improved spatial electronic uniformity in F-incorporated materials, accompanied by suppressed excessive structural transformations during cycling. By integrating chemical regulation at the anion level with microstructural control, this work offers a strategy for stabilizing Ni- and Co-free sodium cathodes.

## Results and Discussion

### Identifying a Strategy for Large-Particle P2 Cathode Formation

Conventional Fe–Mn-based P2 sodium cathodes are commonly synthesized as polycrystalline secondary particles assembled from primary grains, while deliberate strategies to promote large-particle formation in layered sodium systems remain comparatively underdeveloped. Polycrystalline microstructures are vulnerable to grain boundary cracking and exhibit high surface reactivity, as widely recognized in layered cathode systems.^25^ Such vulnerabilities may become more pronounced during electrode fabrication under high mass loading (e.g., >10 mg cm^–2^, Figure S1), where particle fracture can expose fresh surfaces, accelerate parasitic interfacial reactions and destabilize the cathode electrolyte interphase.^26,27^ For air-sensitive layered sodium oxides, additional surface degradation from ambient moisture and CO_2_ exposure during storage can further increase impedance and degrade electrochemical performance.^13^

Motivated by these structural and synthetic considerations, we explored strategies for promoting the formation of large particles. A Na-excess route was first examined as an initial strategy to facilitate reaction completion and potentially promote grain growth under Na-rich conditions. Although P2-type layered oxides are stabilized at relatively lower Na contents compared with those of the O3 phases, a transient Na-rich processing environment can facilitate synthesis by compensating for Na volatilization at elevated temperatures and promoting sufficient Na incorporation during solid-state reactions.^28^ In this sense, Na-excess primarily serves to maintain effective reaction stoichiometry and facilitate sodiation kinetics during calcination. However, this approach provides limited control over nucleation–growth pathways, phase selectivity, and final particle morphology. Consistent with this limitation, Na-excess processing produces nonuniform particle sizes and irregular morphologies with excessive residual Na salts on particle surfaces after washing, as shown in Figure S2 for Fe–Mn–Ti P2 (FMT) cathode materials. Neutron diffraction (ND) analysis (Figure S3) indicates coexistence of P2 with dominant P3, and HAADF-STEM imaging (Figure S4) reveals pronounced lattice shrink and distortion. The corresponding selected-area electron diffraction pattern (SAED, Figure S5) contains discontinuous diffraction rings with discrete spots, indicating the coexistence of multiple crystallographic orientations and limited long-range crystallographic coherence. These outcomes indicate that the Na-excess environment alone does not reliably deliver a pure P2 phase with controlled particle size, morphology, and microstructure.

We therefore introduced an F-assisted molten-salt strategy to regulate phase evolution and crystallization behavior. As indicated by the phase diagram in Figure S6, Na_2_CO_3_, and NaF form a low-melting eutectic mixture, which further enhances ionic transport and promotes sodiation kinetics during calcination.^29^ The molten-salt environment improves mass transport and homogenizes local reaction conditions, while fluorine incorporation modifies the chemical environment during crystal growth, as discussed below. Because the extent of fluorine incorporation, local stoichiometry, and associated electronic effects are inferred from multiple techniques with different probing depths and assumptions, no single measurement can uniquely define the exact chemical state. Instead, ICP-based charge balance, X-ray absorption measurements, TXM analysis, and DFT calculations collectively support a consistent picture in which limited but meaningful fluorine incorporation is accompanied by modest redistribution of TM charge compensation and altered structural evolution. Detailed elemental and phase compositions of F-free (FMT-wo.F) and F-incorporated FMT (FMT-w.F) samples are listed in Table 1.

**Table 1:** Comparative Chemical Compositions, Phase Selectivity, and Particle Morphology of F-Free (FMT-wo.F) and F-Incorporated (FMT-w.F) Samples^a^

	formula	phase composition	morphology	particle size
FMT-wo.F	Na_0.769_Fe_0.254_Mn_0.661_Ti_0.084_O_2_	80% P3 + 20% P2	irregular	nonuniform
FMT-w.F	Na_0.593_Fe_0.259_Mn_0.664_Ti_0.077_O_2_F_0.13_	P2	plate-like	uniform (15–20 μm)

a Na, Fe, Mn, and Ti ratios were obtained from the ICP-MS analysis. The listed formulas should therefore be regarded as nominal compositions rather than as uniquely determined stoichiometries. For FMT-w.F, the fluorine content was estimated from charge neutrality using the measured cation ratios and XANES-constrained TM valence information, with Fe and Ti treated as nominal Fe^3+^ and Ti^4+^ cations, respectively, and assuming two O^2–^ per empirical formula unit. Because the absolute oxygen content was not independently determined, the resulting F_0.13_ value is a model-dependent, charge-balance-derived estimate rather than a directly measured F stoichiometry or crystallographic lattice-F occupancy. The listed empirical composition represents the overall chemically heterogeneous sample and is not intended to be the stoichiometric formula of a single crystalline phase. For FMT-woF, the raw ICP Na signal (Table S1) can include residual sodium salts and therefore does not directly represent the lattice Na content. Complementary F K-edge XAS, ToF-SIMS, TXM-XANES, and DFT analyses support fluorine incorporation/distribution and associated electronic structure trends but do not independently determine a unique chemical formula. Phase compositions were determined from diffraction analysis, while the particle morphology and size were evaluated from SEM observations.

### Structural Integrity and Spatial Distribution of F in NaF-Assisted FMT

The specific crystal structure and phase composition of the F-incorporated Fe–Mn–Ti P2 material, denoted as FMT-w.F, were examined by neutron diffraction (ND). As shown in Figure 1a, Rietveld refinement supports a well-ordered P2 layered phase, with no secondary phases detected within the instrumental resolution. The refined lattice parameters are consistent with a stable P2 structure and indicate that fluorine incorporation does not disrupt the layered structure.

**Figure 1:**
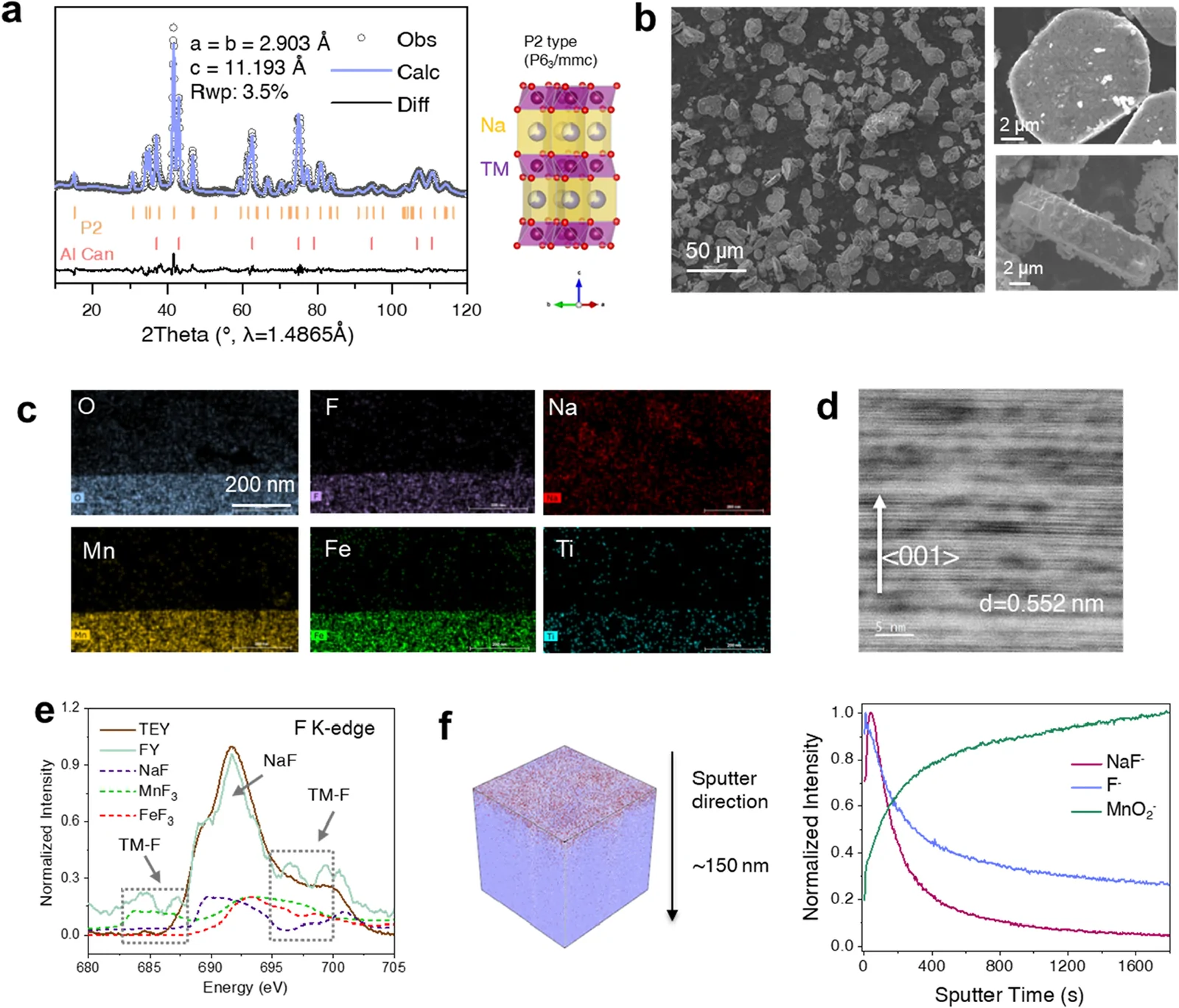
Structural, morphological, and chemical characteristics of F-incorporated Fe–Mn–Ti P2 cathode materials. (a) Rietveld-refined neutron diffraction pattern and corresponding crystal structure model confirm formation of a well-ordered P2 layered phase without detectable secondary phases. (b) SEM images show uniform plate morphology with lateral dimensions of tens of micrometers and dense interiors. (c) Cross-sectional TEM and EDS mapping reveal continuous distributions of Na, Mn, Fe, Ti, O, and F at the nanoscale. (d) High-resolution TEM image resolves lattice fringes along the ⟨001⟩ direction with an average interlayer spacing of approximately 0.552 nm. (e) F K-edge XAS collected in TEY and FY modes show distinct spectral features between surface-sensitive (TEY) and subsurface-sensitive (FY) measurements. (f) Overlaid 3D renderings of key time-of-flight secondary-ion fragments (NaF^–^ in red and total F^–^ in blue) and depth-resolved intensity profiles of NaF^–^ (red), total F^–^, and MnO_2_^–^ fragments reveal near-surface NaF enrichment and a gradual decrease of F signal toward the interior.

Scanning electron microscopy (SEM) images (Figure 1b) reveal a plate morphology with lateral dimensions on tens of micrometers and an average thickness of ∼2 μm. Elemental distributions were investigated by energy-dispersive X-ray spectroscopy (EDS). As shown in Figure S7, Mn, Fe, Ti, and O, are uniformly distributed within individual particles, whereas Na and F are slightly enriched at the surface. Cross-sectional TEM after focused-ion beam (FIB) preparation further confirms uniform TM elemental distribution at the nanoscale and that F is detectable throughout the particle (Figure 1c).

High-resolution HAADF-STEM imaging of the particle interior (Figure 1d) resolves clear lattice fringes along the layered stacking direction, with an interlayer spacing of ∼0.552 nm, consistent with the P2 structure.^6,30^ The corresponding selected-area electron diffraction pattern (SAED, in Figure S8) exhibits a well-defined array of discrete diffraction spots that can be indexed to the P2 structure along the [001] zone axis, indicating a high degree of crystallographic orientation coherence within the selected region. Together with the uniform plate morphology observed by SEM, these observations suggest that the particles possess predominantly large single-crystal-like character with extended coherent crystalline domains, rather than consisting of polycrystalline aggregates.

Soft X-ray absorption spectra (XAS) provide insights into the chemical environment of fluorine (Figure 1e). F K-edge spectra collected in total electron yield (TEY) and fluorescence yield (FY) modes suggest depth-dependent F chemical environments. The surface-sensitive TEY spectrum indicates contributions consistent with NaF-like environments, whereas the subsurface-sensitive FY spectrum suggests additional contributions consistent with TM-F interactions. Depth-resolved TOF-SIMS analysis (Figure 1f) reveals a gradual decrease in fluorine signal intensity from the surface toward the interior rather than a sharp surface-confined profile, indicating that fluorine is not restricted to a simple surface layer. In particular, the NaF signal decreases rapidly within the surface-enriched region, whereas the F- and TM-F-related signals remain detectable at greater sputtering depths. This behavior suggests a transition from a Na–F-rich surface environment to a lower fluorine concentration within the particle interior. Complementary F 1s XPS measurements before and after ion etching further support the presence of different fluorine chemical environments at the surface and in the subsurface region (Figure S9).

From Table 1, the F_0.13_ value for FMT-w.F is a charge-balance-derived estimate based on the ICP cation ratios and XAS-constrained transition-metal valence, assuming two O^2–^ per empirical formula unit. Because the absolute oxygen content was not independently determined, this value should not be interpreted as a directly measured F stoichiometry or F occupancy at the crystallographic O sites. The listed empirical composition represents the overall chemically heterogeneous sample and is not intended to be the stoichiometric formula of a single crystalline phase. It may include contributions from surface-enriched NaF-like species and, therefore, should not be interpreted as lattice fluorine occupancy. Similarly, the interior F^-^/O^-^ ratio from TOF-SIMS (Figure S10) is an uncalibrated secondary-ion intensity ratio and is used only to describe the relative depth distribution of fluorine, not to derive an absolute fluorine stoichiometry. The F_0.03_ composition used in the DFT calculations is therefore a representative dilute F model rather than an experimentally measured interior fluorine content. The negative formation energy (Table S2) indicates that dilute F substitution is energetically feasible in the idealized P2 model but does not determine the actual F site or occupancy.

Overall, F K-edge XAS, TOF-SIMS, and depth-dependent XPS support a gradient-like fluorine distribution with NaF-like species enriched near the surface and lower but persistent fluorine-related environments in the interior. The exact fractions of surface- and lattice-associated fluorine remain unresolved.

### NaF-Assisted Electronic Regulation and Redox Homogenization

To understand the electronic structure differences associated with the NaF-assisted synthesis and the resulting fluorine-related chemical environments, Mn K-edge XANES spectra were collected for F-incorporated FMT (FMT-w.F) and F-free (FMT-wo.F) samples. As shown in Figure 2a, FMT-w.F exhibits a small shift of the rising edge and white-line position toward lower energy relative to FMT-wo.F, consistent with a slightly lower average Mn oxidation state. Both samples maintain Mn valence predominantly between 3+ and 4+. To be specific, by comparing the E_0.5_ position of Mn_2_O_3_ and MnO_2_, the calculated Mn average valence in FMT-w.F and FMT-wo.F is 3.694+ and 3.766+, respectively. DFT calculations shown in Figure S11 further support the possibility that dilute F incorporation can promote a modest increase in Mn^3+^-like character within the simplified model system. We acknowledge that the computational models simplify the experimental system, which contains heterogeneous surface and interior fluorine environments, different Na contents, and distinct P2/P3-phase compositions. The calculations were therefore not intended to reproduce all of the experimental compositional and structural details or to directly prove the full experimental stabilization mechanism. Instead, they were used to isolate the possible intrinsic effect of dilute F incorporation on the local Mn electronic structure under the controlled model conditions. To minimize the influence of the Na stoichiometry, the Na content was fixed at 0.59 in both computational models. For the F-free model, the P3 structure was selected because P3 is the dominant phase in the experimental FMT-wo.F sample. The Mn magnetic moments, Bader net charges, and local structural distortions were subsequently evaluated by using these simplified models. All three descriptors consistently indicate a modest shift toward Mn^3+^-like character in the F-containing model. Fe K-edge XANES spectra (Figure S12) show negligible differences between the two samples, indicating that Fe valence remains approximately unchanged between the two materials.

**Figure 2:**
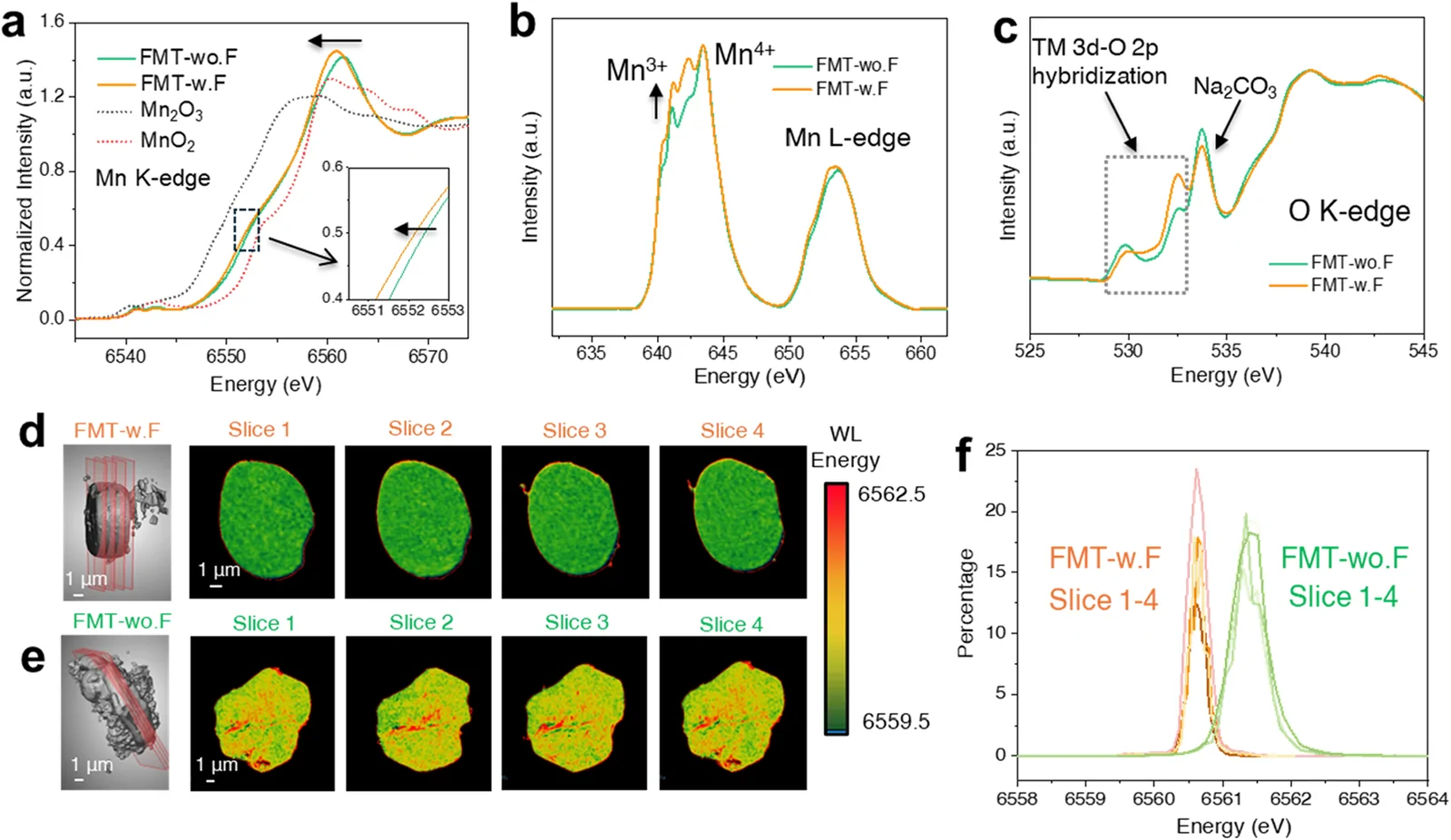
Influence of F incorporation on TM redox behavior and spatial chemical distribution. (a) Mn K-edge XANES spectra of FMT-wo.F and FMT-w.F. (b) Mn L-edge and (c) O K-edge soft XAS comparing FMT-wo.F and FMT-w.F in the TEY mode. 3D-TXM reconstructions of (d) FMT-w.F and (e) FMT-wo.F, based on the Mn K-edge white-line position with the color scale in eV, with four internal cross-sectional slices displayed for each particle. (f) Histograms of Mn K-edge white-line energies extracted from the TXM slices from (d) and (e). A broader energy distribution seen in FMT-wo.F suggests a more heterogeneous Mn valence distribution.

On the basis of the ICP-derived cation ratios and charge-balance analysis constrained by the approximate XANES-derived transition-metal valence estimates, the charge-balance-based nominal compositions of FMT-w.F and FMT-wo.F are reported as Na_0.593_Fe_0.259_Mn_0.664_Ti_0.077_O_2_F_0.13_ and Na_0.769_Fe_0.254_Mn_0.661_Ti_0.084_O_2_, with F content calculated by using charge compensation. The method-dependent compositions and valence trends are summarized in Table S3. Because the F content and TM valence are evaluated using multiple techniques with different probing depths, sensitivities, and assumptions, the exact chemical stoichiometry cannot be uniquely assigned from any single method.

Surface-sensitive Mn L-edge XAS spectra indicate a higher fraction of Mn^3+^ species near the surface of FMT-w.F compared with that of FMT-wo.F (Figure 2b). In addition, O K-edge spectra exhibit discernible changes in features associated with TM-O hybridization (Figure 2c), suggesting that the NaF-assisted synthesis and associated fluorine-related environments modify the local anion coordination and TM-O electronic interactions. These spectral changes are consistent with local charge redistribution and modified TM-O covalency. However, the O K-edge spectra alone do not provide direct evidence of reversible oxygen redox, and no such assignment is made here.

Three-dimensional transmission X-ray microscopy (3D-TXM) mapping provides spatially resolved insights into Mn valence distribution within individual particles (Figure 2d,e). FMT-w.F displays lower Mn valence and a homogeneous valence distribution across the particle volume, whereas FMT-wo.F exhibits higher Mn valence and more pronounced spatial heterogeneity. Statistical analysis of Mn valence histograms confirms a relatively homogeneous distribution and lower average Mn valence in FMT-w.F (Figure 2f). These results indicate that the NaF-assisted synthesis is associated with a modified local Mn electronic structure and improved spatial redox homogeneity. However, this difference is interpreted as the coupled outcome of phase composition, crystallographic coherence, particle morphology, and fluorine-related chemical environments rather than as an isolated effect of lattice fluorine incorporation.

### Enhanced Electrochemical Durability and Stability in Ambient Air Conditions

Electrochemical measurements demonstrate improved performance for the F-incorporated material. Under a voltage window of 1.5–4.3 V, FMT-w.F delivers a higher reversible discharge capacity of ∼172 mAh g^–1^ compared with ∼151 mAh g^–1^ of FMT-wo.F (Figures S13 and S14). FMT-w.F also exhibits enhanced cycling stability over 50 cycles compared to FMT-wo.F, maintaining a higher capacity retention (88% vs 86%).

When cycled within 1.5–4.0 V, FMT-w.F also shows improved initial discharge capacity (∼140 mAh g^–1^) and significantly enhanced cycling performance (87% retention over 240 cycles) compared with FMT-wo.F (∼135 mAh g^–1^ and 70%). As shown in Figure 3a, FMT-w.F maintains stable Coulombic efficiency and reduced capacity decay. Galvanostatic charge–discharge profiles (Figure 3b) show more stable voltage evolution with lower polarization and reduced voltage hysteresis during cycling.

**Figure 3:**
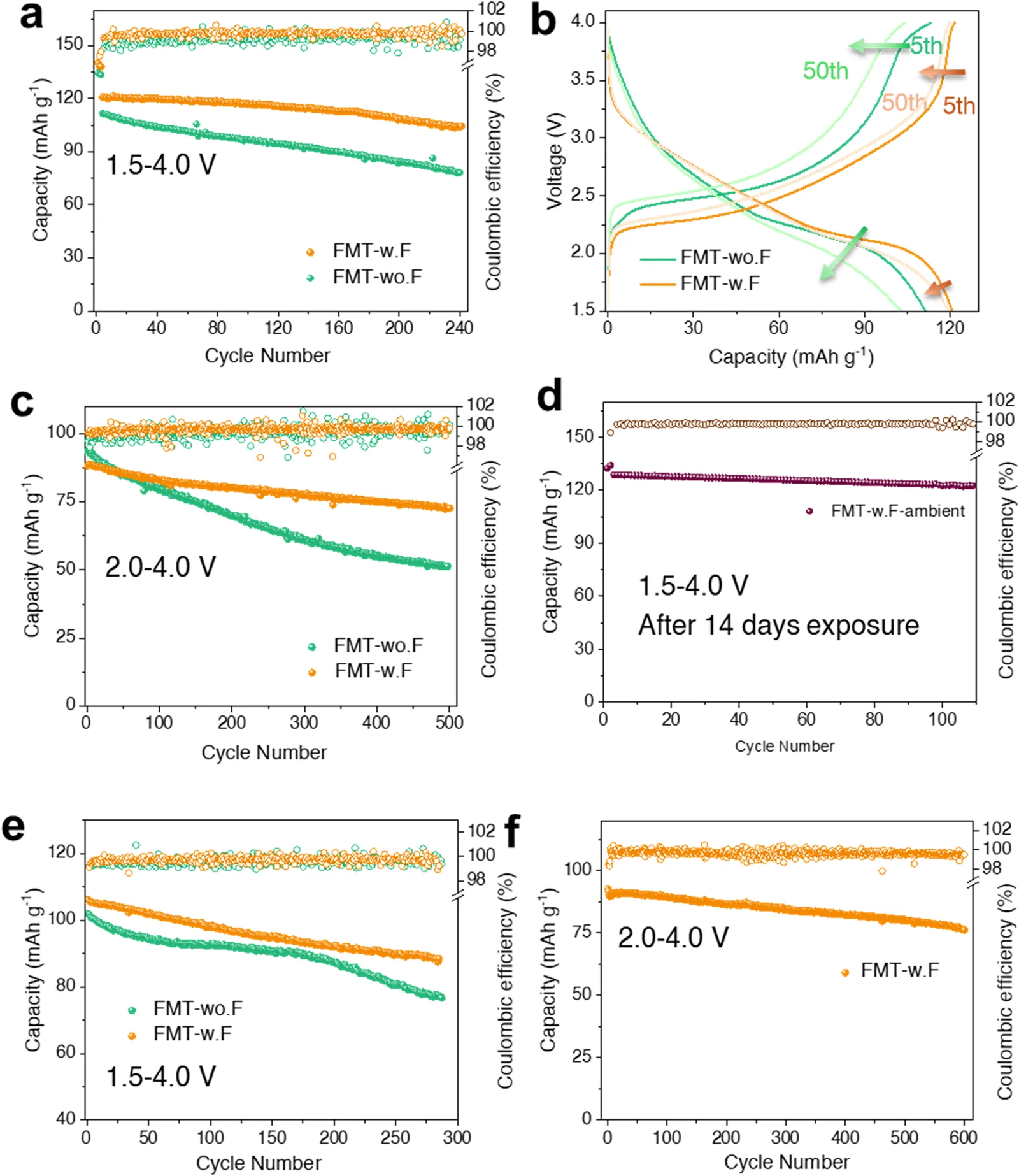
**I**mpact of F incorporation on the electrochemical performance of FMT at 25 °C. (a) Cycling performance FMT-wo.F and FMT-w.F at 0.5C (1C = 130 mA g^–1^) within 1.5–4.0 V vs Na/Na^+^, with the initial two formation cycles at 0.1C, with a cathode active material mass loading of ∼5 mg cm^–2^. (b) Charging and discharging curves of selected cycles of FMT-wo.F and FMT-w.F at 0.5C within 1.5–4.0 V. For each sample, the 5th and 50th cycles are shown. (c) Long-term cycling of FMT-wo.F and FMT-w.F at 0.5C within 2.0–4.0 V. (d) Cycling performance of FMT-w.F within 1.5–4.0 V at 0.5C after 14 days of ambient storage, with the initial two formation cycles at 0.1C. (e) Full-cell cycling of FMT-wo.F and FMT-w.F paired with hard carbon at 0.5C within 1.5–4.0 V, with a cathode active material mass loading of ∼10 mg cm^–2^. (f) Long-term full-cell cycling of FMT-w.F at 0.5C within 2.0–4.0 V. All cells were tested at 25 °C, with the electrolyte composed of 1 M NaPF_6_ in diglyme.

For extended cycling tests (Figure 3c), FMT-w.F maintains ∼82% capacity retention after 500 cycles compared with 55% for FMT-wo.F. A comparison with representative recently reported Ni/Co-free P2-type sodium layered oxide cathodes under half-cell conditions further demonstrates the competitive overall performance of FMT-w.F, particularly its balance of reversible capacity and long-term cycling stability, while differences in voltage windows, current densities, and cycling protocols should be considered (Table S4).

To evaluate ambient-storage stability more rigorously, FMT-w.F and FMT-wo.F were exposed to identical ambient conditions for 14 days (∼50% humidity) and subsequently characterized in parallel. Both samples retain comparable electrochemical performance relative to their pristine counterparts, with no appreciable capacity deterioration after storage (Figures 3d and S15). SEM images reveal particulate deposits on both particle surfaces after exposure, indicating that air-induced surface reactions still occur, although they do not cause measurable electrochemical degradation under the present conditions. The relatively large-particle morphology shared by both samples likely limits the overall extent of surface reactions with ambient H_2_O and CO_2_.^31^ Surface-enriched NaF-like species in FMT-w.F may provide an additional contribution to interfacial stabilization, but their independent effect cannot be isolated from particle size and morphology effects in the present comparison.

In full-cell configuration paired with hard carbon anodes, FMT-w.F continues to outperform FMT-wo.F within the 1.5–4.0 V window (Figure 3e), delivering higher reversible capacity (∼106 mAh g^–1^ vs ∼102 mAh g^–1^) and improved retention (83% vs 75% over 280 cycles). Ultralong cycling stability is achieved for this Ni-, Co-, and Li-free sodium cathode system, with ∼83% capacity retention after 600 cycles (Figure 3f).

An additional F-free NaCl/Na_2_CO_3_ molten-salt control was also evaluated to distinguish the contribution of molten-salt fluxing from fluorine-related effects. Although this F-free molten-salt treatment promotes the growth of relatively large particles, the resulting material remains phase-mixed and exhibits inferior reversible capacity and cycling stability (Figure S16). These results indicate that molten-salt-assisted particle growth alone is insufficient to reproduce the phase selectivity and electrochemical performance of FMT-w.F.

### Structural and Redox Evolution during Electrochemical Cycling

As shown in Figure 4a, FMT-wo.F exhibits pronounced peak shifts and intensity variations during the first discharge and subsequent charge processes, indicating a severe structural rearrangement. For example, the (101) peak undergoes a large shift accompanied by noticeable intensity attenuation, indicating substantial changes in lattice parameters and reduced structural coherence during cycling. Meanwhile, the (012) peak disappears during the middle stage of discharge and reappears upon charging, suggesting a temporary disruption of the corresponding long-range ordering rather than simple solid-solution behavior. In addition, the opposite intensity evolution of the (104) peak in the gray region relative to the (012) peak indicates stacking rearrangement and redistribution of diffraction intensity during cycling. To further evaluate these changes, the positions of the reliably resolved (101), (012), (104), and (110) reflections were fitted when observable, and the corresponding lattice parameters were estimated (Figure S17a,b). Because of the low signal-to-noise ratio, severe peak overlap, and temporary peak disappearance, reliable multiphase refinement could not be performed for FMT-wo.F. The fitted values are therefore treated as semiquantitative indicators of structural evolution.

**Figure 4:**
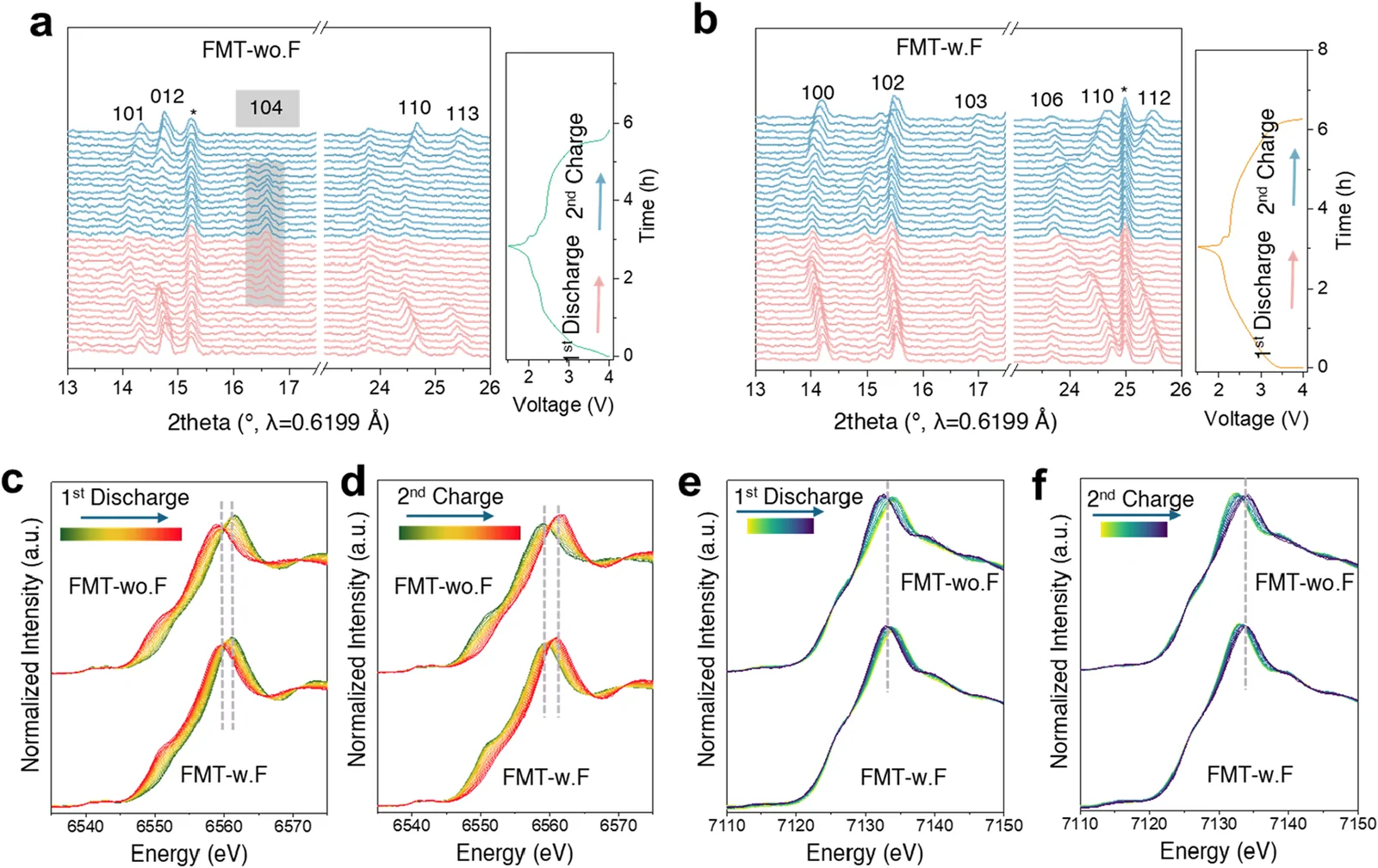
Structural and redox evolution during electrochemical cycling. *In situ* XRD patterns and corresponding voltage profiles during the first discharge and second charge at 0.33C within 1.5–4.0 V for (a) FMT-wo.F and (b) FMT-w.F. The peaks marked by * originated from coin cell parts. In situ Mn K-edge XANES during (c) the first discharging and (d) second charging processes, and in situ Fe K-edge XANES during (e) the first discharging and (f) second charging processes, as the cells were also tested at 0.33C during 1.5–4.0 V.

In contrast, FMT-w.F shows smoother and more-reversible structural evolution (Figure 4b). The overall peak evolution, such as (100), (102), and (103) peaks, remains more coherent than that in FMT-wo.F, indicating that the parent layered phase is better-preserved during cycling. Peak position and lattice parameter fitting further show more continuous changes and better structural recovery during charge and discharge (Figure S17c,d). However, the reaction is not purely featureless or fully solid solution-like. Peaks assigned to the parent layered phase show moderate and largely reversible shifts during discharge and charge, consistent with lattice breathing associated with Na extraction and reinsertion.^32^ At the same time, several peaks exhibit transient splitting and the temporary appearance of additional peaks, indicating short-lived coexistence of closely related layered phases and limited structural rearrangement during cycling. These changes are substantially less disruptive than those observed in FMT-wo.F, where a stronger intensity redistribution and more pronounced peak evolution indicate greater structural heterogeneity. Together, these results suggest that fluorine incorporation does not eliminate phase evolution but instead constrains it to a more-reversible and structurally coherent pathway.

Consistent with the structural observations, *in situ* Mn K-edge XANES spectra show moderate white-line shifts for FMT-w.F, compared with the more pronounced evolution in FMT-wo.F (Figure 4c,d), as well as fitted white-line position in Figure S18. The shifts were referenced to the corresponding pristine states and are used as relative redox descriptors rather than as precise absolute Mn valences. Both samples exhibit largely reversible Mn K-edge white-line shifts during charge and discharge. FMT-w.F shows a more moderate spectral evolution, consistent with its more-reversible structural response. Fe K-edge XANES shows only minor changes for FMT-w.F but more noticeable evolution for FMT-wo.F near the end of discharge and charge (Figures 4e,f and S19), indicating stronger local coordination changes in the F-free material.

Notably, FMT-w.F delivers a higher reversible capacity than FMT-wo.F over long-term cycling. At the same time, FMT-wo.F exhibits larger redox and structural transformations during initial cycling, indicating that a broader apparent redox response does not necessarily translate to more efficient reversible Na storage. This behavior suggests a larger fraction of charge compensation in FMT-wo.F may proceed through less-reversible structural rearrangement, spatially heterogeneous reaction, or parasitic high-voltage processes, which can lead to accelerated degradation over long-term cycling. In contrast, the more reversible structural and redox evolution of FMT-w.F correlates with its lower polarization and improved capacity retention.

Because oxygen redox has been increasingly reported in P-type layered Na cathodes, it is possible that anionic charge compensation also contributes to the electrochemical behavior of both materials.^33^ Although FMT-w.F delivers a higher reversible capacity with a smaller measured Mn redox response, this observation alone is insufficient to establish enhanced reversible oxygen redox. Without direct oxygen-sensitive probes or gas-evolution analysis, we do not assign the relative oxygen redox contribution here. Future studies will be needed to determine whether fluorine incorporation changes the extent, reversibility, or degradation pathways of the oxygen redox.

### F Incorporation Enhances Particle Integrity upon Electrochemical Cycling

Postcycling cross-sectional SEM images reveal extensive cracking and internal damage in the FMT-wo.F particles (Figure 5a). Severe intraparticle defects are observed upon magnification, including pronounced deformation bands, localized bending, and internal cracking within individual particles, as shown in Figure S20. HAADF-STEM imaging further reveals particle exfoliation and widespread crack distribution, as shown in Figure 5b. In enlarged views (Figure 5c), clear local lattice distortion and interlayer inhomogeneity are detected, including local expansion regions with a *d*-spacing of ∼0.807 nm, accompanied by tearing features. In addition, intragranular cracks throughout the particle interior are identified, as shown in Figure S21. These observations indicate substantial structural breakdown and residual structural heterogeneity in the F-free material after prolonged cycling. In contrast, the F-incorporated particles retain their platelet morphology with substantially reduced crack formation. FMT-w.F shows an intact morphology after cycling, without major cracks inside (Figures 5d and S22). Elemental mapping confirms that F and other elements remain uniformly distributed within the particle after cycling, as shown in Figures 5e and S23, with fluorine retained within the structure rather than being largely depleted from the bulk. High-resolution imaging of the particle interior shows clear lattice fringes corresponding to the (002) planes, with a *d*-spacing of ∼0.526 nm, as shown in Figure 5f, indicating preservation of the layered structure. Cycled XRD (Figure S24) further shows that the sample with F incorporation evolves more reversibly during electrochemical cycling. The main layered peak P2 (002) exhibits moderate shifts during Na^+^ extraction and intercalation, consistent with reversible changes in interlayer spacing. In contrast, FMT-wo.F shows more pronounced redistribution and residual structural heterogeneity in P3 (003) peak after cycling, indicating less-reversible phase evolution.

**Figure 5:**
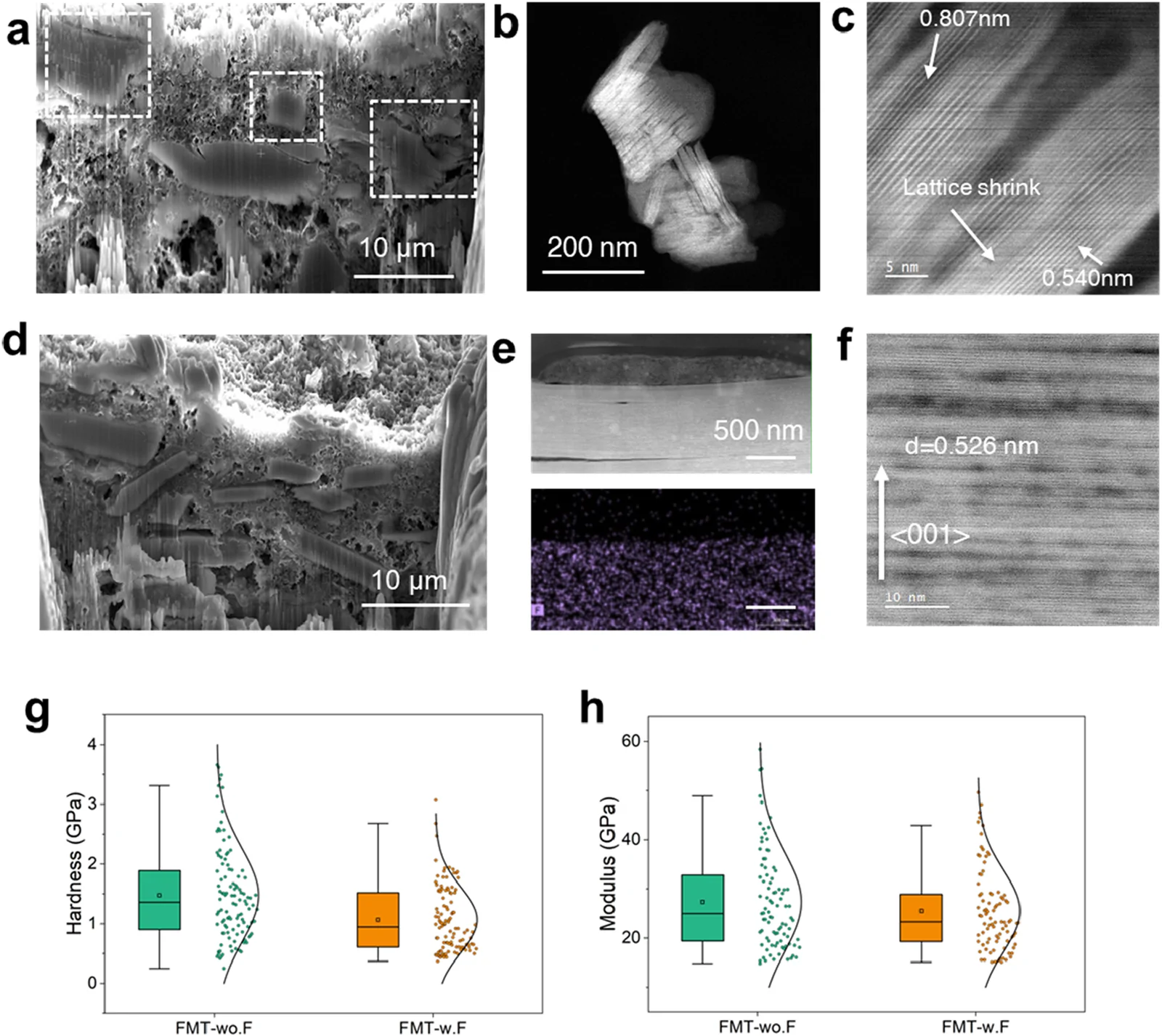
Postcycling morphological evolution and mechanical property evaluation. (a) Cross-sectional SEM image of FMT-wo.F after 100 cycles showing extensive internal cracking and structural collapse. (b) Low-magnification HAADF-STEM of cycled FMT-wo.F revealing particle exfoliation and widespread crack distribution. (c) High-magnification HAADF-STEM of cycled FMT-wo.F showing local lattice distortion, inhomogeneity of interlayer spacing. (d) Cross-sectional SEM image of FMT-w.F after 100 cycles showing preserved particle morphology with minimal visible cracking. (e) Cross-sectional low-magnification HAADF-STEM with F EDS elemental mapping of cycled FMT-w.F. (f) High-magnification HAADF-STEM image of cycled FMT-w.F showing well-defined layered structure similar to the pristine state in Figure 1d. Nanoindentation measurements of pristine FMT-wo.F and FMT-w.F particles showing (g) hardness and (h) elastic modulus distributions.

Because crack generation is closely related to intrinsic mechanical properties, nanoindentation measurements were performed on pristine samples to evaluate chemomechanical characteristics, as shown in Figure 5g. The results show no major statistical differences in elastic modulus and hardness between F-incorporated and F-free particles in their pristine state. However, postcycling microscopy reveals substantially reduced crack formation in FMT-w.F relative to the F-free counterpart, as discussed earlier. Thus, we propose that the impact of F incorporation resides mostly in improving structural reversibility and thus long-term electrochemical durability rather than in mechanics in the pristine state. Accordingly, the improved morphological integrity is interpreted as part of the coupled outcome of P2 phase selectivity, crystallographic coherence, redox homogeneity, and fluorine-related chemical environments produced by the NaF-assisted synthesis rather than as an isolated effect of lattice fluorine incorporation.

## Summary

Stabilizing Fe–Mn-based P2 sodium cathodes requires coordinated control of structural reversibility, chemomechanical stability, and air stability to improve electrochemical performance. In this work, we show that NaF-assisted molten-salt synthesis enables such coordinated control. By coupling a Na-rich crystallization environment with molten-salt fluxing and fluorine-related chemical regulation, this approach promotes a reliable formation of large Fe–Mn–Ti Na-layered cathode particles with high P2 phase selectivity. Fluorine incorporation reshapes how the material evolves, moderates TM redox states, and mitigates irreversible structural transformations during cycling, leading to enhanced chemomechanical stability and battery cycling stability in half- and full-cells. The improved battery performance results from the combined effects of P2 phase selectivity, crystallographic coherence, redox homogeneity, and fluorine-related chemical environments induced by the NaF-assisted synthesis rather than from lattice fluorine incorporation alone. Further work should clarify how incorporating foreign anions affects oxygen redox and chemomechanical evolution during cycling. Overall, the strategy presented here shows that anion-enabled regulation can help design mechanically resilient and manufacturing-compatible energy storage materials.

## Supplementary Material



## Notes

Additional SEM, EDS, HAADF-STEM and TEM images; ICP, XRD, ND, XPS, XAS, TOF-SIMS; chemical structures and DFT calculations; supporting figures/tables for the electrochemical data; and comparison of compositions or valence-related information inferred from different experimental and computational methods (PDF)
