# Supplementary material for: Fluorine-Mediated Molten-Salt Crystallization Regulates Phase Evolution and Structural Stability in Sodium Layered Oxides
Source: J Am Chem Soc. 2026 Oct 21;148(43):46666–76. doi: 10.1021/jacs.6c10977 (PMC13741820; doi:10.1021/jacs.6c10977)
Supplement: Supplementary file 1 [file ja6c10977_si_001.pdf]

Fluorine-Mediated Molten-Salt Crystallization Regulates Phase Evolution and Structural Stability in Sodium Layered Oxides

Chenguang Shi[1], Junyi Yao[2], Xiaowen Zhan[2*], Jeongok Choe[3], Xixian Yang[3], He Zhou[2], Amir Ashraf Elnashar[3], Rupayan Ghosh[1], Callum Connor[2], Anika Tabassum Promi[1], Weibo Huang[2], Hongyu Wang[4], Sooyeon Hwang[5], Kim Kisslinger[5], Si Athena Chen[6], Dali Yang[7], Lu Ma[7], Hui Zhong[7], Jianming Bai[7], Xianghui Xiao[7], Dennis Nordlund[8], David Reed[9], Xiaolin Li[9], Kejie Zhao[3], Feng Lin[1,2*]

1 Department of Chemistry, Virginia Tech, Blacksburg, Virginia 24061, United States

2 School of Engineering, Brown University, Providence, Rhode Island 02912, United States

3 School of Mechanical Engineering, Purdue University, West Lafayette, IN 47907, United States

4 Institute for Critical Technology and Applied Science, Virginia Tech, Blacksburg, Virginia 24061, United States

5 Center for Functional Nanomaterials, Brookhaven National Laboratory, Upton, NY, 11973, United States

6 Neutron Scattering Division, Oak Ridge National Laboratory, Oak Ridge, TN 37830, United States

7 National Synchrotron Light Source II, Brookhaven National Laboratory, Upton, NY, 11973, United States

8 Stanford Synchrotron Radiation Light Source, SLAC National Accelerator Laboratory, Menlo Park, CA 94025, United States

9 Energy and Environmental Directorate, Pacific Northwest National Laboratory, Richland, WA 99352, United States

Corresponding authors: Xiaowen_zhan@brown.edu; feng_lin@brown.edu

Experimental information

Materials Synthesis

Both FMT-wo.F and FMT-w.F cathodes were synthesized by solid-state reactions. For FMT-wo.F, 0.8M Na_2CO_3 (Sigma-Aldrich, 99.95-100.05%), stoichiometric Fe_2O_3 (Thermo Scientific Chemicals, 99.9%), Mn_2O_3 (Thermo Scientific Chemicals, 98%), TiO_2 (Sigma-Aldrich, 99-100.5%) were used as starting materials. For FMT-w.F, a mixed sodium source consisting of 0.6M Na_2CO_3 and 0.4M NaF (ACS, 99.0%) was employed, while maintaining the same stoichiometric Fe_2O_3, and Mn_2O_3 and TiO_2. The starting powders were ball-milled for 10 h at 400 rpm, pressed into pellets, and calcined in a box furnace (Lindberg, model: BF51894C-1) in air at 900 °C for 12 h, with a heating rate of 5 °C min^{-1}. After cooling to room temperature, the calcined products were ground and washed three times with cold deionized water, followed by one rinse with isopropyl alcohol. The solids were collected by centrifugation and subsequently recalcined at 500 °C for 3 h. After cooling to 200 °C, the products were ground again and transferred into an Ar-filled glovebox for electrode fabrication and characterization. For the fluorine-free molten-salt control, NaF was replaced with an equimolar amount of NaCl, while the amount of Na_2CO_3, total salt-to-precursor ratio, precursor composition, ball-milling procedure, calcination conditions, water-washing process, and drying treatment were kept identical to those used for FMT-w.F.

Materials characterizations

Synchrotron X-ray diffraction (XRD) was collected at 7-BM (QAS) (NSLS-II, Brookhaven National Laboratory), using a PerkinElmer area detector. XRD patterns were continuously collected during the electrochemical process, with each scan acquired in 30 s. Two-dimensional XRD images from the detector were integrated and converted using PyFAI with appropriate masks on individual images. For ex-situ XRD, samples were measured at 28-ID-2 (NSLS-II, Brookhaven National Laboratory). Phase fractions and lattice parameters were refined using GSAS-II software.

Synchrotron X-ray absorption spectroscopy (XAS) was also performed at beamline 7-BM (QAS) (NSLS-II, Brookhaven National Laboratory). Sequential Fe K-edge and Mn K-edge XAS spectra (30 s per scan) were collected continuously during heating.

Neutron diffraction (ND) was conducted on the HB-2C WAND^2 beamline at the high flux isotope reactor (HFIR), Oak Ridge National Laboratory. A Ge (113) monochromator with a take-off angle of 51.5° provided a neutron wavelength of 1.4865 Å. Neutron diffraction patterns were collected at room temperature over a 2theta range of 10°-120°. Neutron diffraction data were reduced in Mantid software. Phase fractions and lattice parameters were refined using GSAS-II software.

Soft X-ray absorption spectroscopy was performed at beamline 8-2 at the Stanford Synchrotron Radiation Lightsource. Measurements were conducted at room temperature under ultrahigh vacuum (~10^{-9} torr) using both total electron yield and fluorescence yield modes. The energy resolution was 0.2 eV with a beam spot of 1 mm^2.

Soft XAS spectra were analyzed using PyMca.

The XANES-3D transmission X-ray microscopy (TXM) was performed at beamline 18-ID (FXI) (Brookhaven National Laboratory). Reconstruction of TXM datasets was performed using TXM_Sandbox.[1] The segmentation, quantification and visualization of the TXM results were performed with dragonfly v.2022.2.

Time-of-Flight Secondary Ion Mass Spectrometry (ToF-SIMS) analyses were carried out using an ION-TOF TOF.SIMS 5-100 instrument (ION-TOF USA INC., Chestnut Ridge, NY, USA) at the Surface Analysis Facility at the University of Delaware. A pulsed 30 keV BiMn cluster ion gun was used as the primary ion source. A low-energy electron flood gun was applied for neutralizing surface charge, and a dual Cs/O_2 ion source dedicated to depth profiling operations. Depth profiling was performed using a dual-beam interlaced mode, using a 500 eV Cs^+ ion beam over 300 × 300 μm^2 area, and a 30 keV Bi^{3+} ion beam for data collection over a central 100 × 100 μm^2 analysis region, with a spatial resolution of 256 × 256 pixels. The sputtering rate was calibrated at ~0.08 nm s^{-1} by a SiO_2 reference. Negative secondary ions were monitored and collected. Data was analyzed using SurfaceLab software (version 7.3).

Morphology was examined using scanning electron microscopy (JEOL IT-500HR) at 5 kV. High-angle annular dark-field scanning transmission electron microscopy (HAADF-STEM) and energy dispersive X-ray spectroscopy (EDS) were performed on Thermo Fisher Scientific Talos F200X and Hitachi HD2700C microscopes at 200 kV at the Center for Functional Nanomaterials (Brookhaven National Laboratory).

For inductively coupled plasma mass spectrometry (ICP-MS) analysis, ~2 mg sample was dissolved in aqua regia composed of concentrated hydrochloric acid and nitric acid in a 3:1 volume ratio for 12 h. 1 mL of the acid mixture was diluted with 39 mL of deionized water. The prepared samples were analyzed using a Thermo Scientific iCAP RQ ICP-MS instrument to quantify the Na and TM concentrations.

Mechanical Characterization Nanoindentation (Keysight G200) tests are performed in an argon-filled glovebox (O_2 and H_2O < 0.5 ppm). Berkovich indenter is used for the CSM (Continuous Stiffness Measurement) experiments with an indentation depth of 80 nm to measure the modulus, and hardness. Hardness H and elastic modulus E are determined using a Berkovich tip. The elastic modulus and Poisson ratio of the diamond indenter (Ei,vi) are 1141 GPa and 0.07, respectively.

Electrochemical characterizations

Composite cathodes were prepared by mixing active material, acetylene carbon black, and poly(vinylidene fluoride) binder in N-methyl-2-pyrrolidone at a mass ratio of 80:10:10. The slurry was cast onto carbon-coated aluminum foil and dried under vacuum at 120 °C overnight. Circular electrodes with a diameter of 10 mm were punched and transferred into an Ar-filled glovebox, with mass loading of ~ 5 mg cm^{-2} or ~10 mg cm^{-2}.

CR2032 coin cells were assembled using sodium metal or presodiated hard carbon as the anode. Glass microfiber separators (Whatman GF/D) were employed. The electrolyte was 1 M $NaPF_6$ dissolved in diglyme, with 70 µL added per cell.

Galvanostatic charge and discharge tests were carried out at the selected voltage range at 25 °C using a multichannel battery tester (Wuhan Land). Cycling was performed at 0.5 C, where 1 C corresponds to 130 mA g^{-1}. Cells for in-situ XRD and XAS tests were running at 0.33 C.

Theoretical calculation

First-principles calculations were carried out in Vienna Ab-initio Simulation Package (VASP). Atomic model of P3 phase FMT was constructed from a 3 × 3 × 1 supercell contains 88 atoms, corresponding to the composition $Na_{0.59}Mn_{0.63}Fe_{0.26}Ti_{0.11}O_2$. P2 phase FMT model was built using a 4 × 4 × 1 supercell, with a random oxygen atom being substituted by fluorine atom. The model includes 115 atoms with the composition of $Na_{0.59}Mn_{0.625}Fe_{0.25}Ti_{0.125}O_{1.97}F_{0.03}$. To reduce the number of simultaneously varying compositional factors, the Na content was fixed at 0.59 in both the F-containing P2 and F-free P3 models. This controlled treatment excludes differences in Na-vacancy concentration as an additional variable and enables comparison of the local electronic and TM–anion bonding environments at the same Na concentration. Because the models also differ in their P2/P3 stacking configurations, the calculations represent a comparison between phase-relevant model structures rather than a complete isolation of the F contribution.

Projector-augmented-wave (PAW) pseudopotentials were used to describe the ionic cores, and Perdew-Burke-Ernzerhof (PBE) in generalized gradient approximation (GGA) framework was adopted for the exchange correlation effects. DFT+U method was employed to capture the strong Coulomb interactions of localized electrons from transition metals, and the effective U-J values were set to 4.0, 4.0 and 3.5 eV for Mn, Fe and Ti, respectively. The plane wave cutoff energy was set as 520 eV. The Γ-center 2 × 2 × 1 and 2 × 2 × 2 k-point mesh were adopted for P3 and P2 phase, respectively. Electronic self-consistency is achieved when energy change is below 5×10^{-7} eV. Lattice parameters and ionic positions were relaxed until the forces on ions were less than 0.01 eV/Å. Spin polarization is considered.

The F-doping formation energy was evaluated by

$$E_f = E_{FMT+F} - E_{FMT} + 0.5\times E_{O2} - 0.5\times E_{F2}$$

where E_{FMT+F} and E_{FMT} denotes energies of F-doped and pristine FMT, respectively. E_{O2} and E_{F2} are energies of O_2 and F_2. To eliminate the over binding reported for GGA when computing E_{O2} and E_{F2}, we take the calibration values of -1.198 eV and -0.884 eV, respectively, to the molecular reference energies.[2]

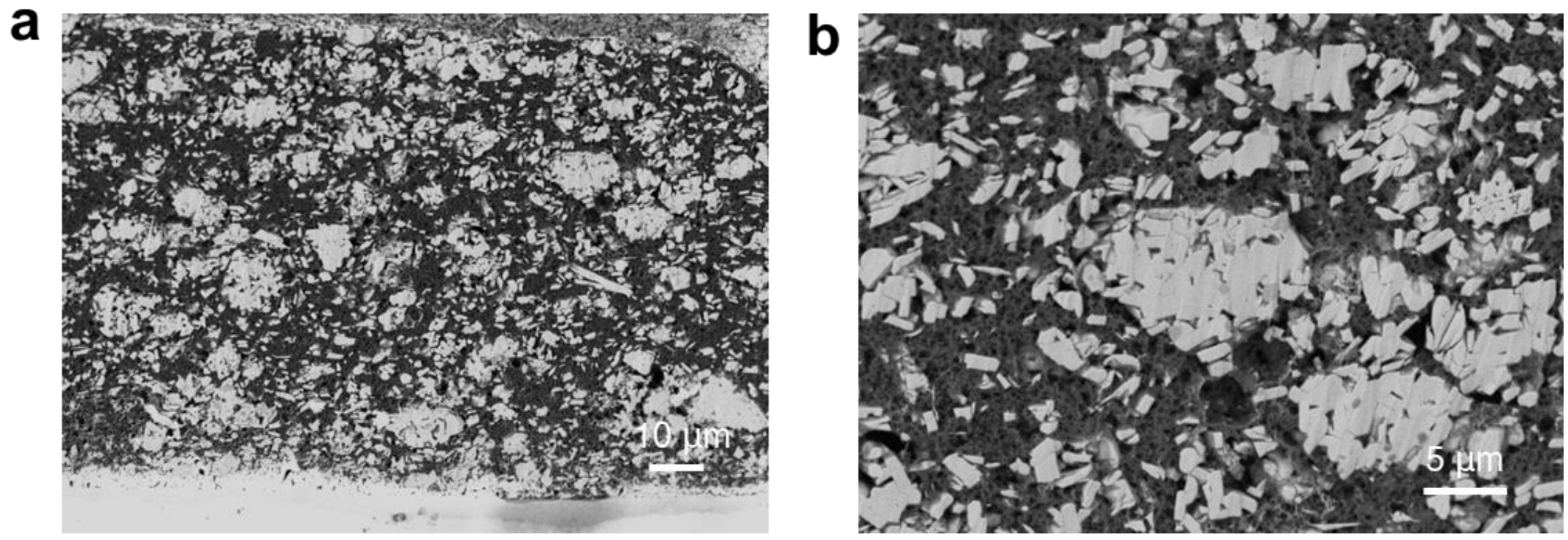

Figure S1. SEM images in different magnifications of high mass loading electrodes (>10 mg cm^{-2}) of P2-type $Na_xFe_{0.25}Mn_{0.65}Ti_{0.10}O_2$ by conventional solid-synthesis method. This shows cracks generation during electrode processing.

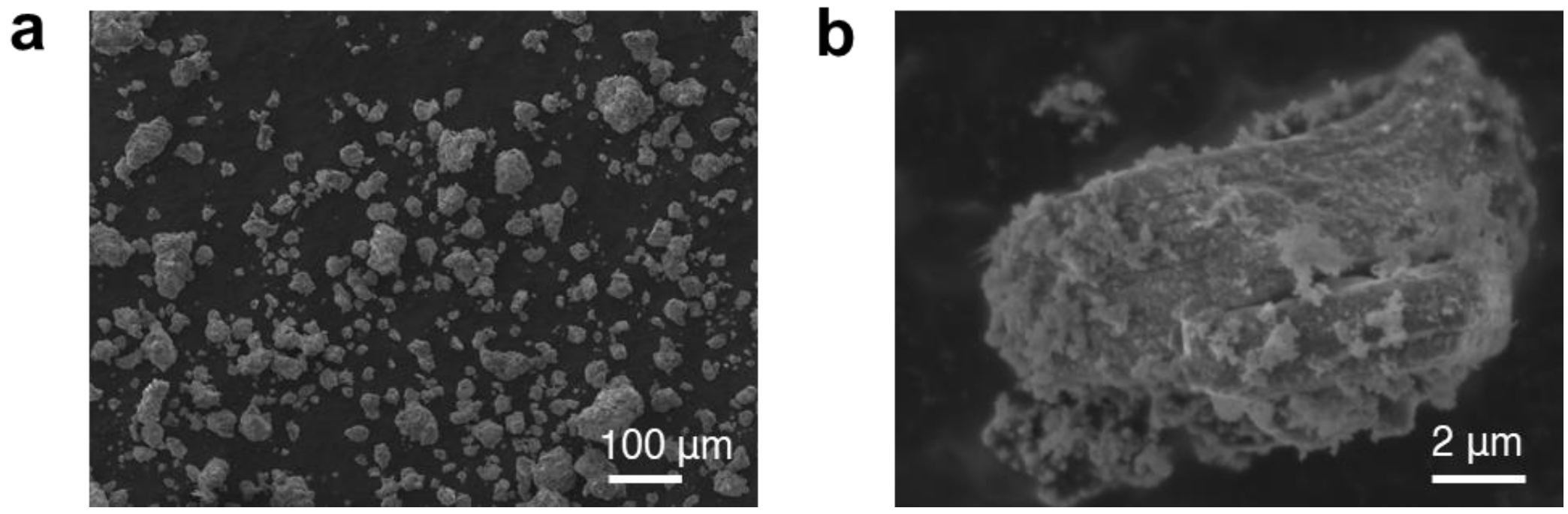

Figure S2. SEM images in different magnifications of FMT ($Na_{0.413}Fe_{0.254}Mn_{0.661}Ti_{0.084}O_2$) synthesized from the approach of using excessive Na_2CO_3 salt (see Experimental Method). This method only results in non-uniform particle size, and without defined a particle morphology, with Na salt residues observed on the particle surface.

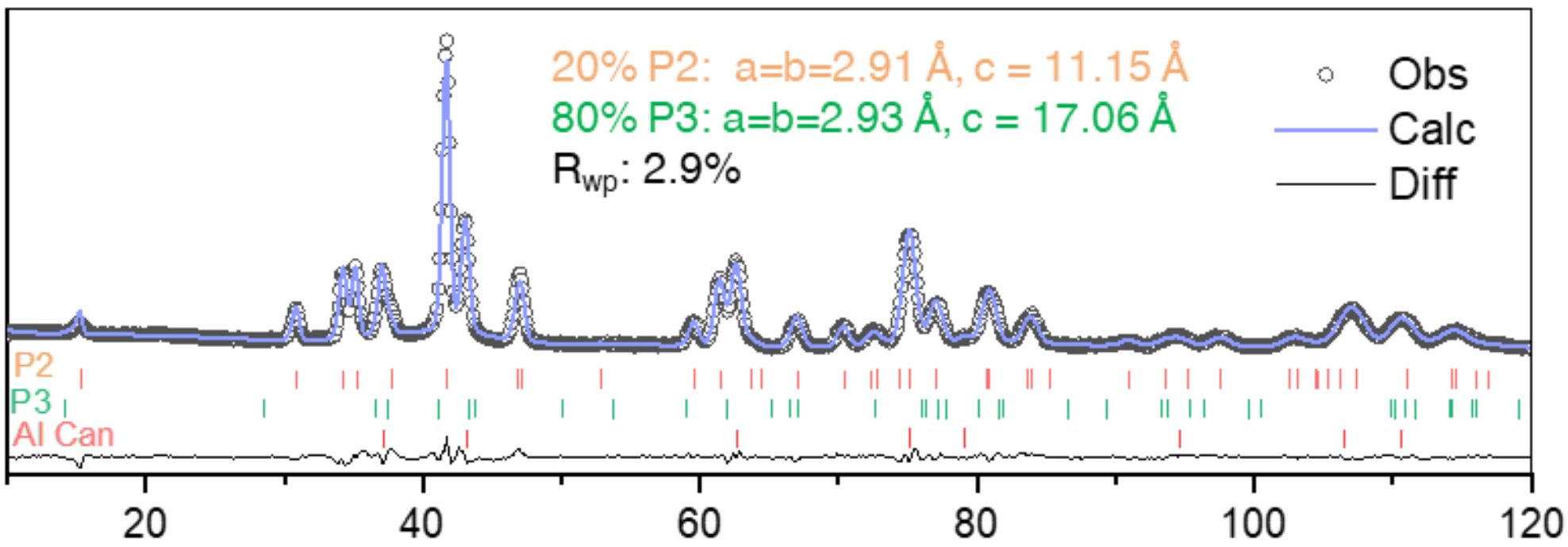

Figure S3. Neutron diffraction (ND) pattern and refinement results of FMT synthesized from the approach of using excessive Na_2CO_3 salt. It consists of P3-dominant phase, as well as P2 phase coexistence.

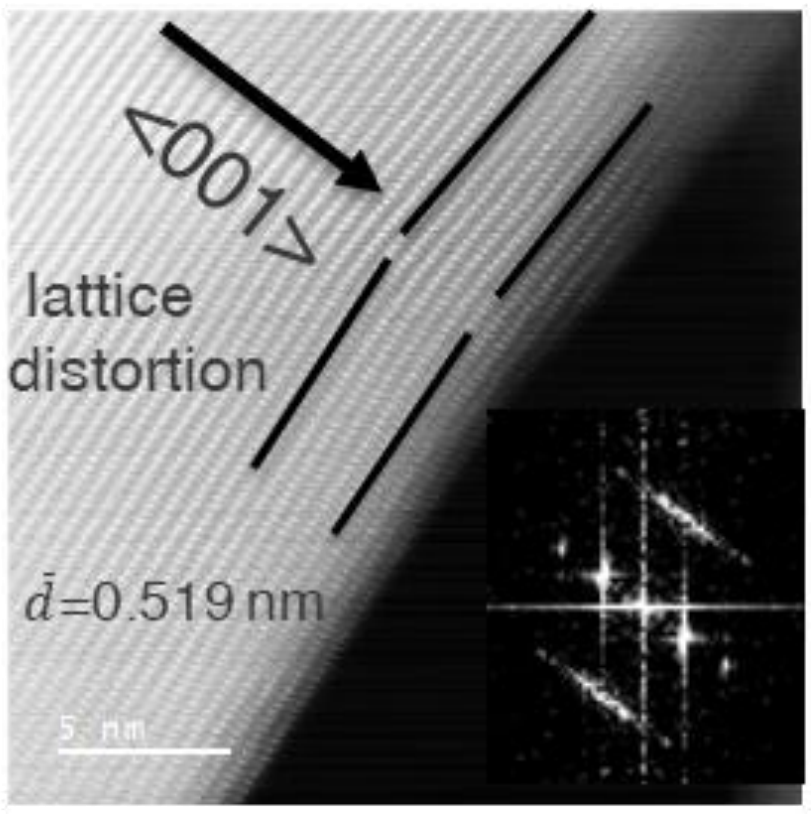

Figure S4. HAADF-STEM image of pristine FMT synthesized from the approach of using excessive Na_2CO_3 salt. It shows severe lattice distortion with a much reduced interlayer distance compared with 0.552 nm of a typical P2 phase.[3]

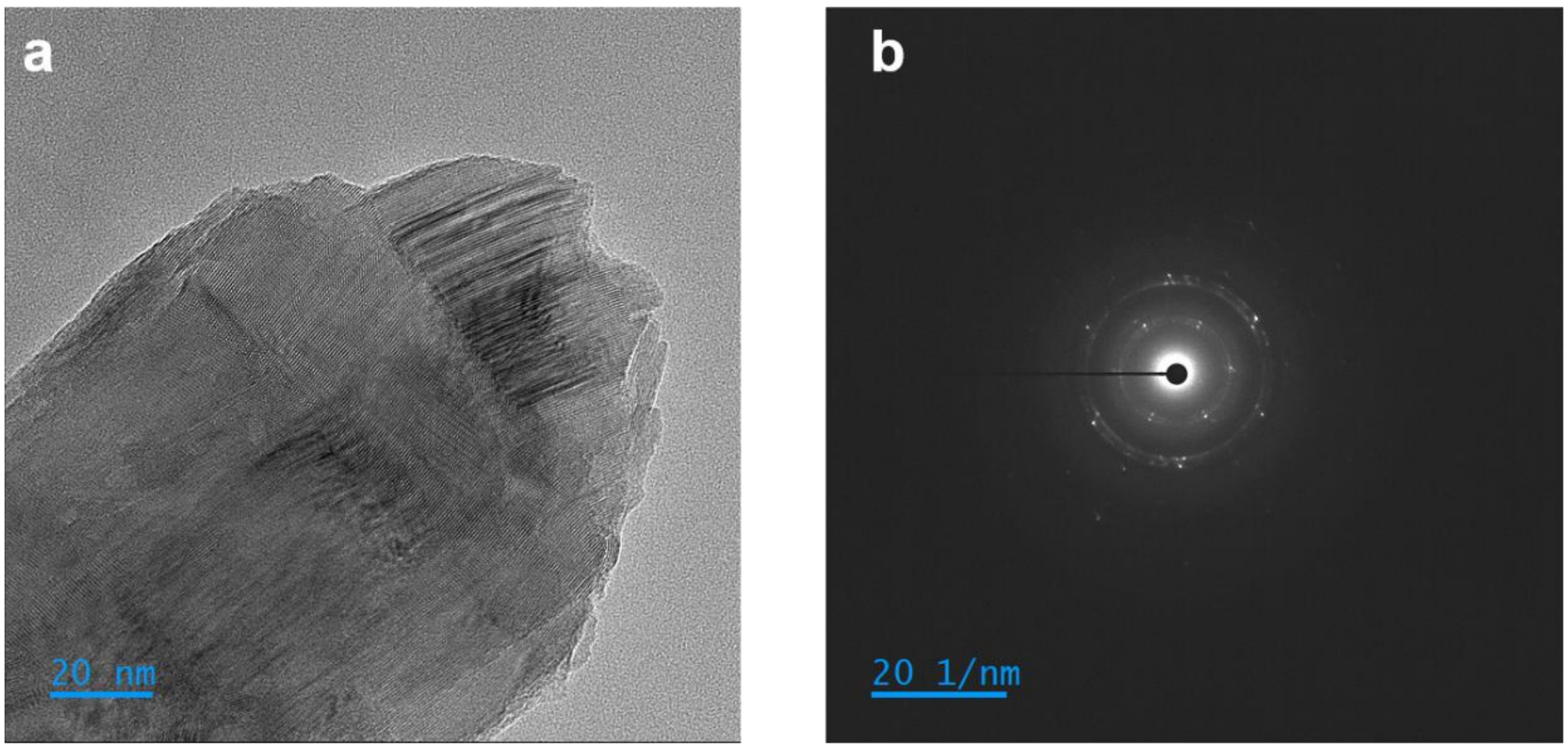

Figure S5. TEM and SAED characterization of FMT-wo.F. (a) TEM image of an FMT-wo.F particle showing multiple overlapping lattice-fringe regions with different orientations, consistent with the presence of several crystalline domains within the observed area. (b) The corresponding SAED pattern exhibits discontinuous diffraction rings composed of discrete spots, indicating a polycrystalline or multidomain character with crystallographic orientation dispersion.

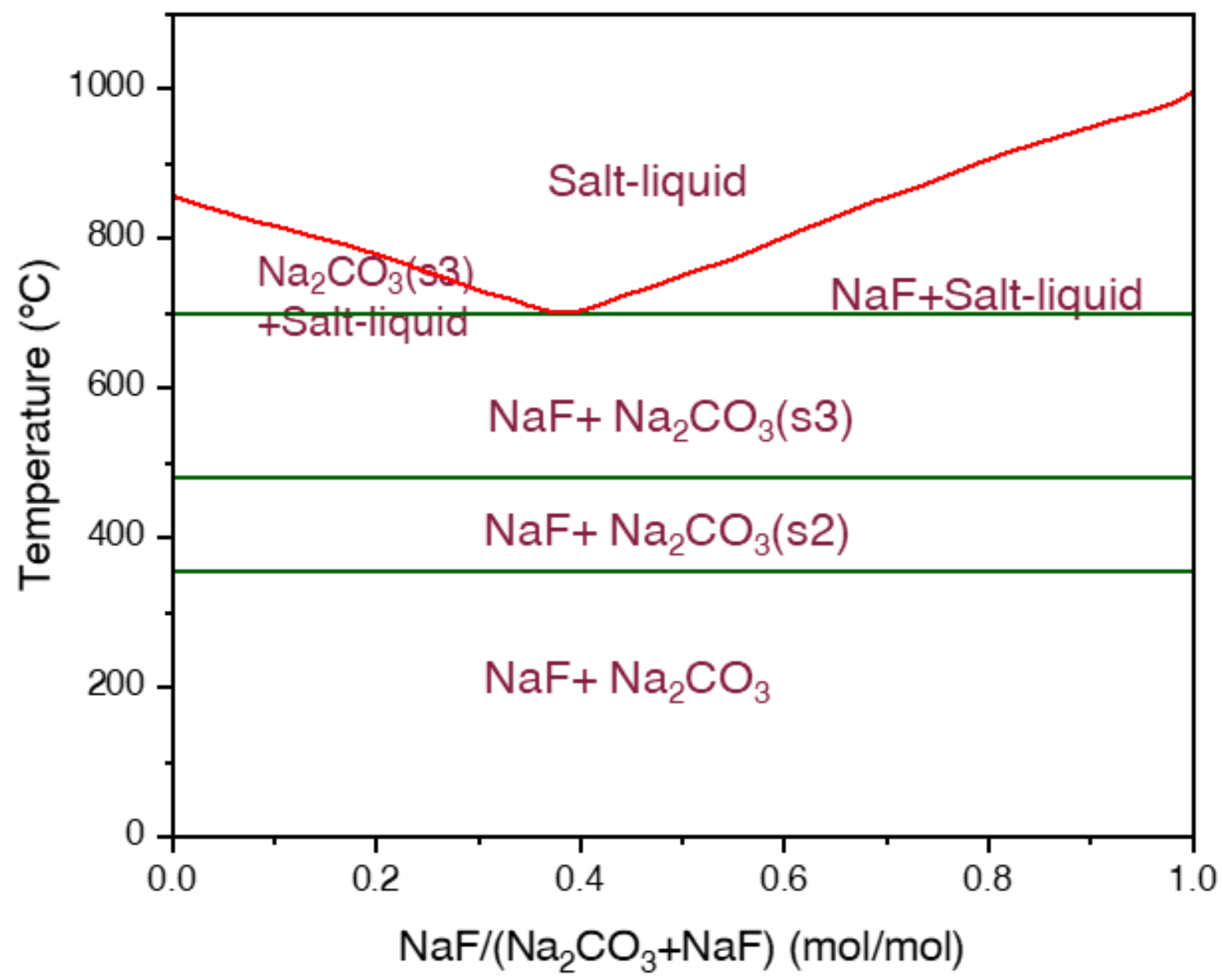

Figure S6. Phase diagram of NaF and Na_2CO_3[4]

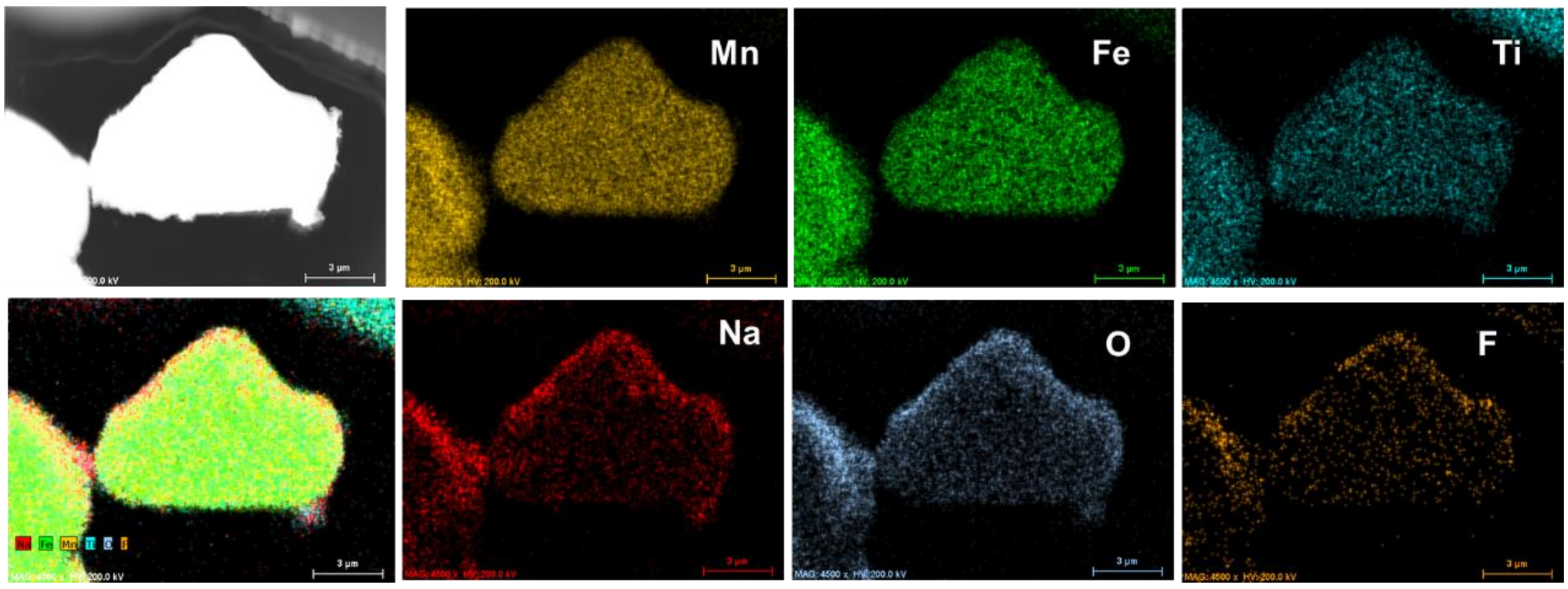

Figure S7. EDS-mapping of FMT-w.F particle. This indicates homogenous distribution of TM cations, and F is enriched at the surface and also present in the interior of the particles.

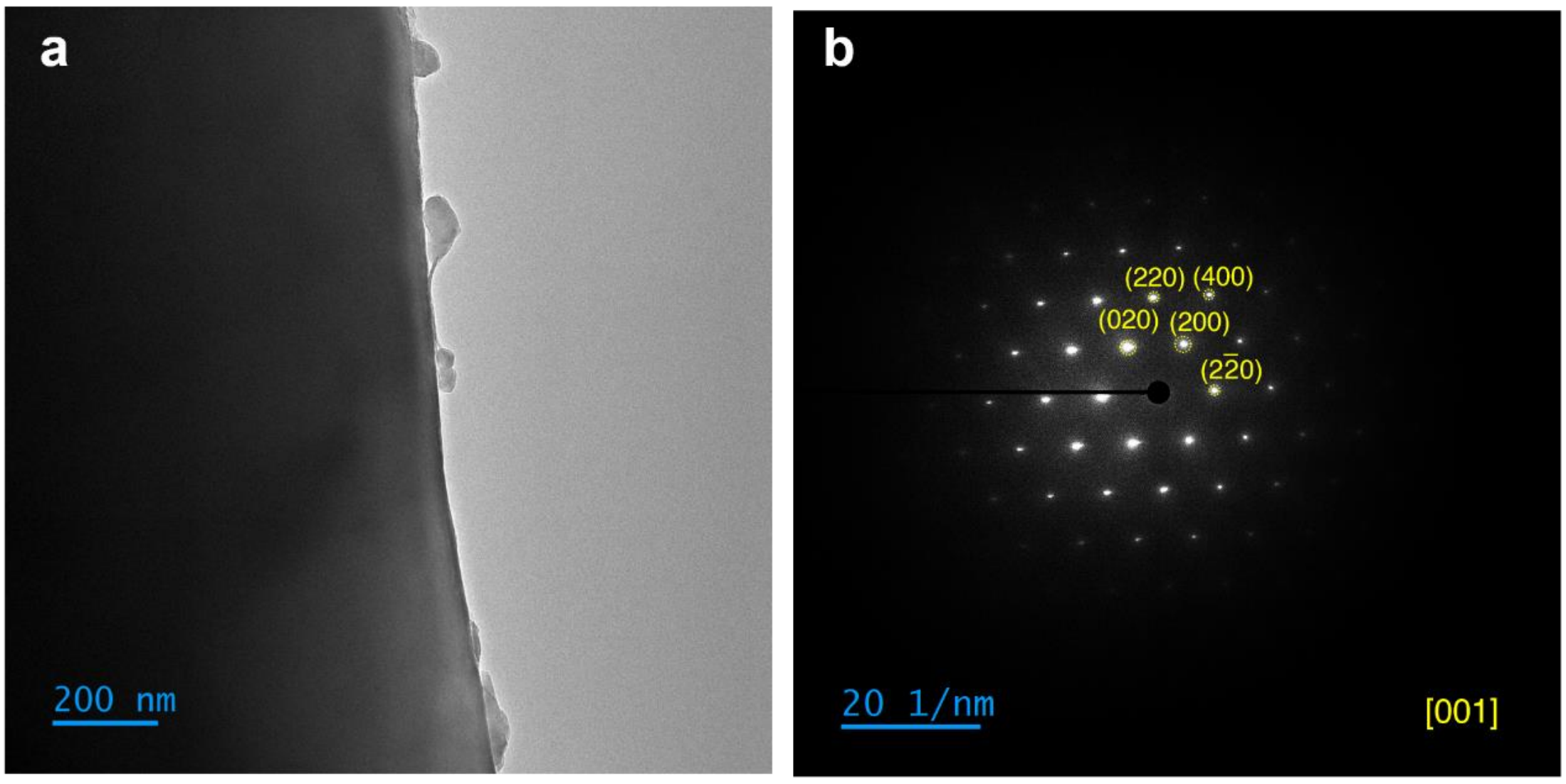

Figure S8. (a) Representative TEM image of FMT-w.F showing the region selected for electron diffraction. (b) Corresponding SAED pattern indexed to the P2 structure along the [001] zone axis, showing a well-defined array of discrete diffraction SAED spots.

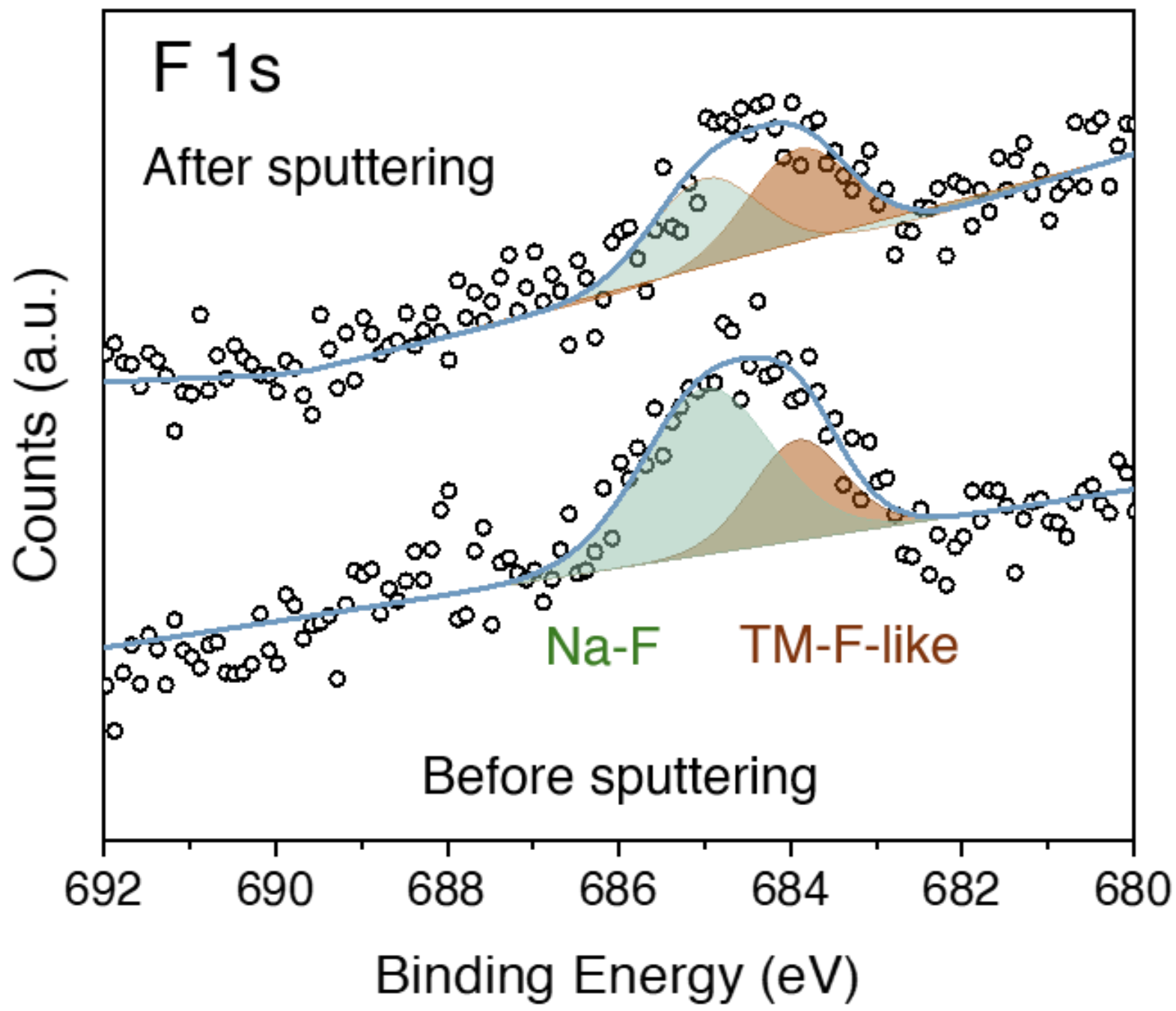

Figure S9. F 1s XPS spectra of FMT-w.F collected from the surface region (bottom) and the sputter-exposed inner region (top). The open circles represent the experimental data, and the blue lines represent the overall fitted curves. The components centered at approximately 685.0 and 683.9 eV are assigned to NaF-like and TM-F-like environments, respectively.[5-7]

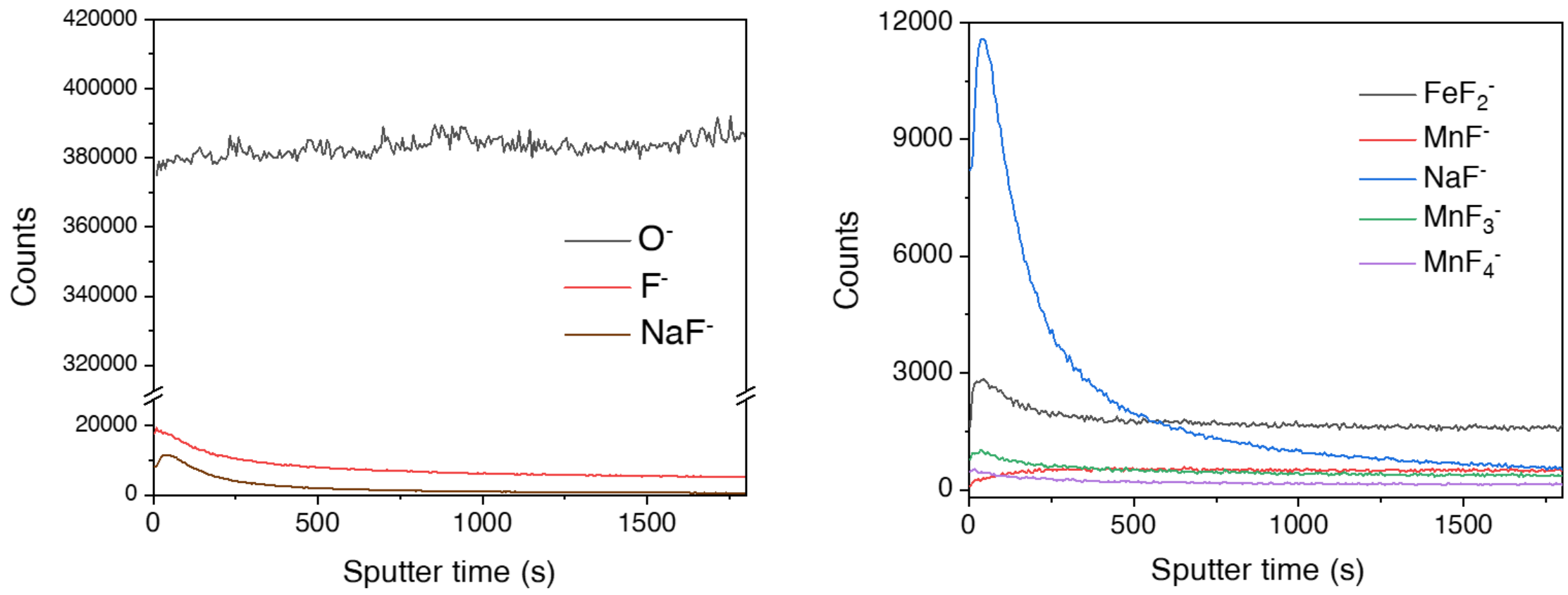

Figure S10. Negative-ion TOF-SIMS profiles of F^- and O^- signals collected during depth sputtering of the fluorinated cathode sample. The fluorine signal decreases gradually from the surface toward the interior, rather than showing a sharp surface-confined profile. These results indicate a surface-enriched and interior-dilute fluorine distribution in FMT-w.F. Because TOF-SIMS ion yields depend strongly on the local chemical matrix and no composition-matched calibration standard was used, the measured intensities should be regarded as semi-quantitative. They do not independently determine the absolute fluorine content or the fraction of lattice-incorporated fluorine.

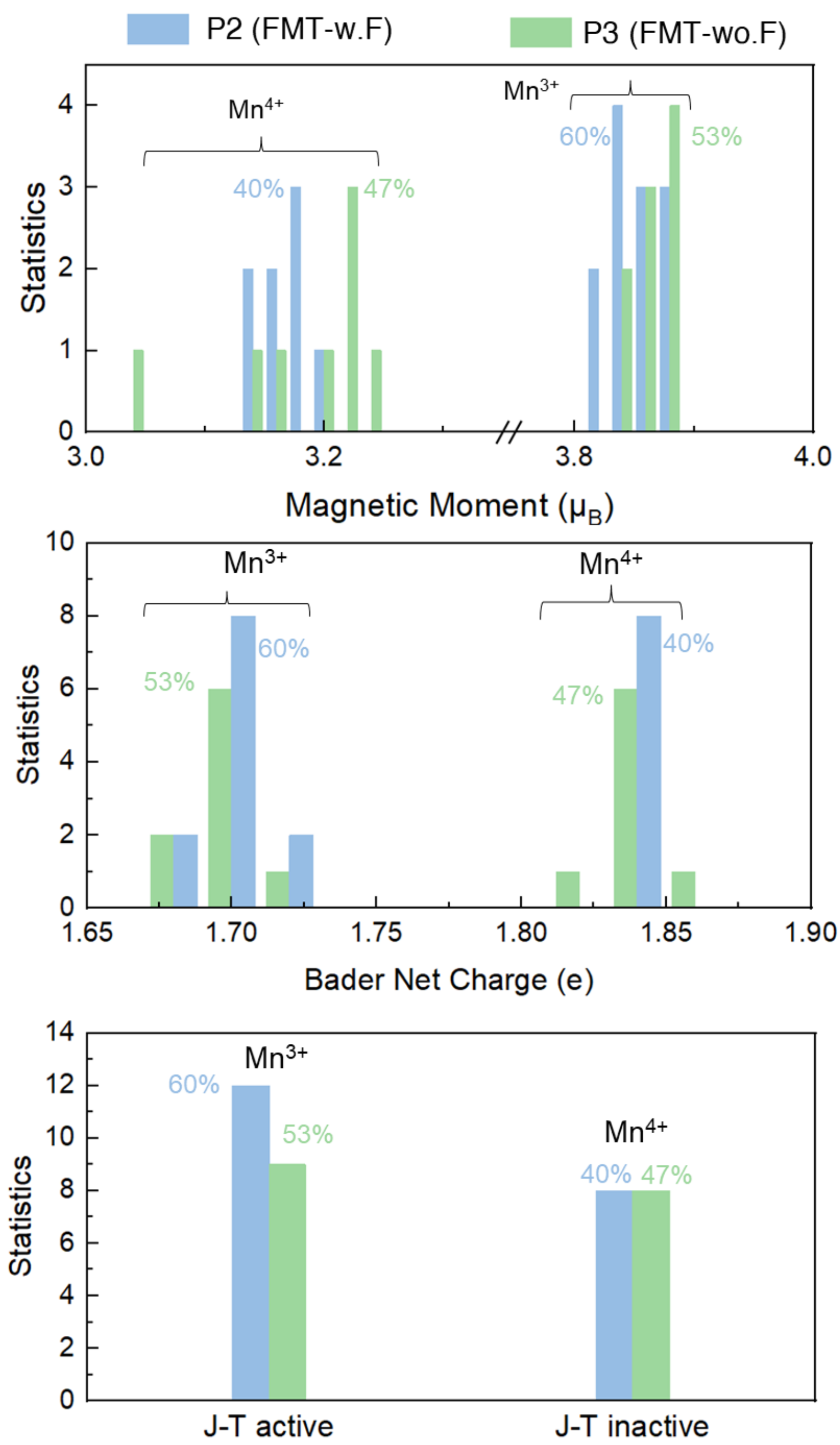

Figure S11. DFT-calculated magnetic moment, Bader net charge, and local structural distortion of Mn in the F-free and F-containing models. To exclude the effect of Na content, all calculations were performed at a fixed Na content of 0.59. The results consistently indicate slightly lower Mn valence in the F-containing structure, suggesting slight Mn reduction upon F incorporation.

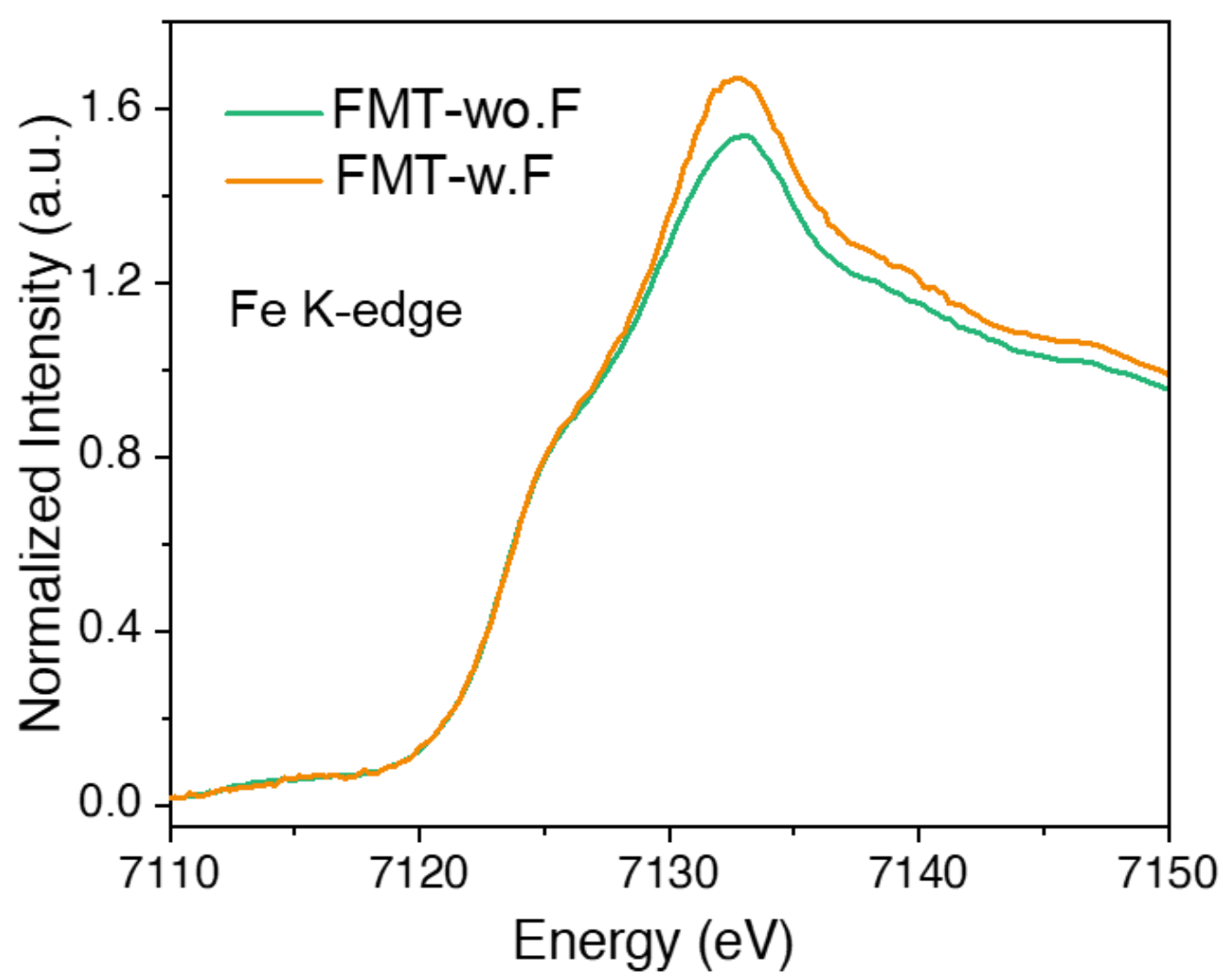

Figure S12. Fe K-edge XANES spectra of FMT-wo.F and FMT-w.F., showing basically the same Fe valence by comparing $E_{0.5}$ position.

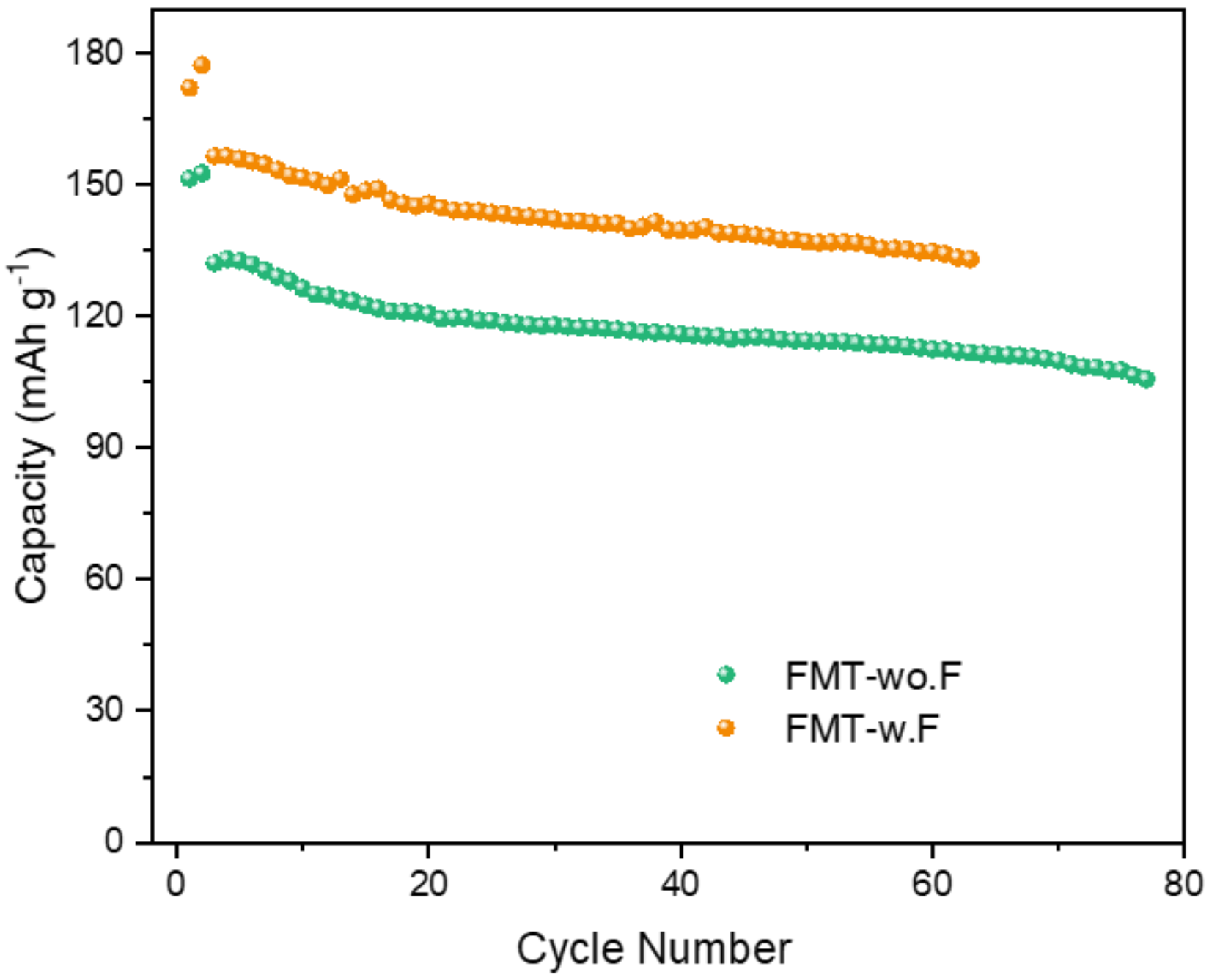

Figure S13. Cycling performance at 0.5 C (1C = 130 mA g^{-1}) between 1.5-4.3 V of FMT-wo.F and FMT-w.F, with a cathode active material mass loading of ~5 mg cm^{-2} and sodium metal as the anode. Electrolyte is composed of 1M $NaPF_6$ in diglyme.

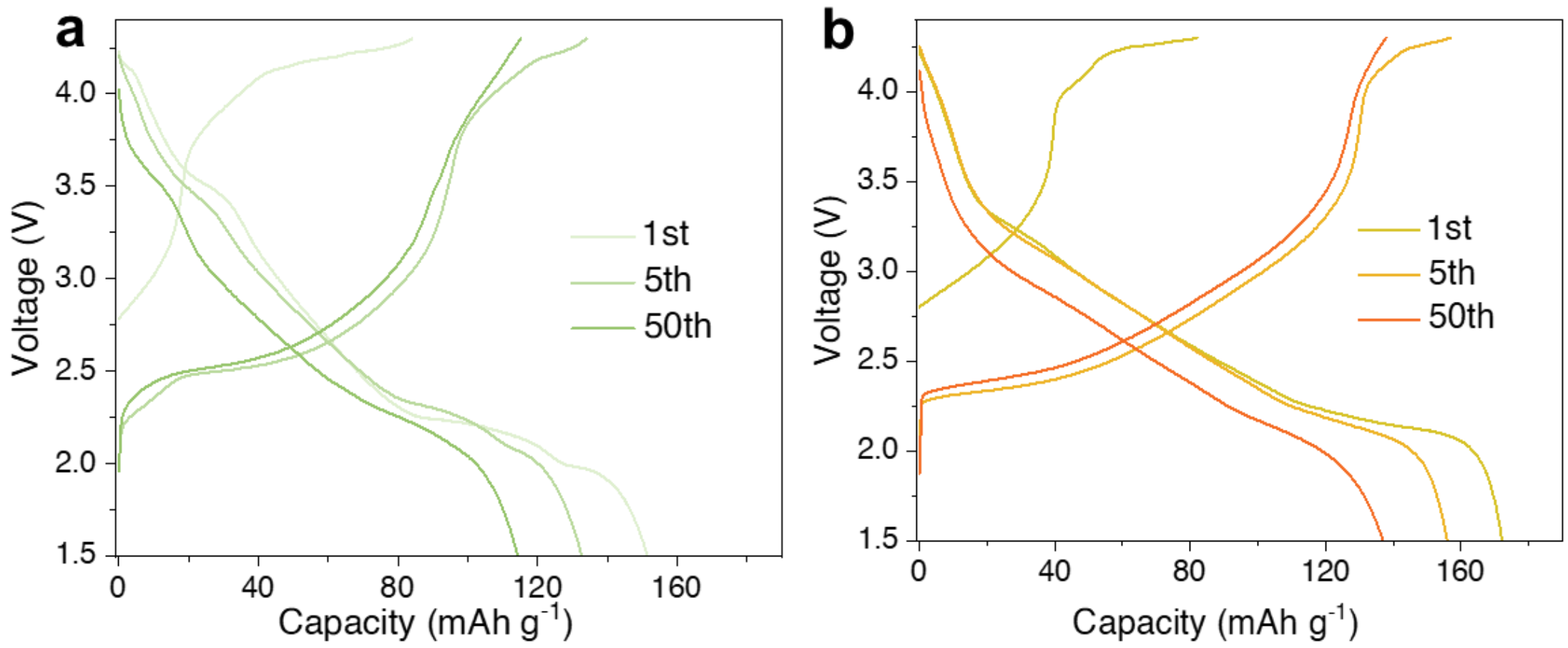

Figure S14. Galvanostatic charge-discharge curves at 0.1 C for 1st cycle and 0.5 C for 5th and 50th cycles in the voltage range of 1.5-4.3 V for (a) FMT-wo.F and (b) FMT-w.F. All cells were tested at 25 °C, with the electrolyte composed of 1M $NaPF_6$ in diglyme.

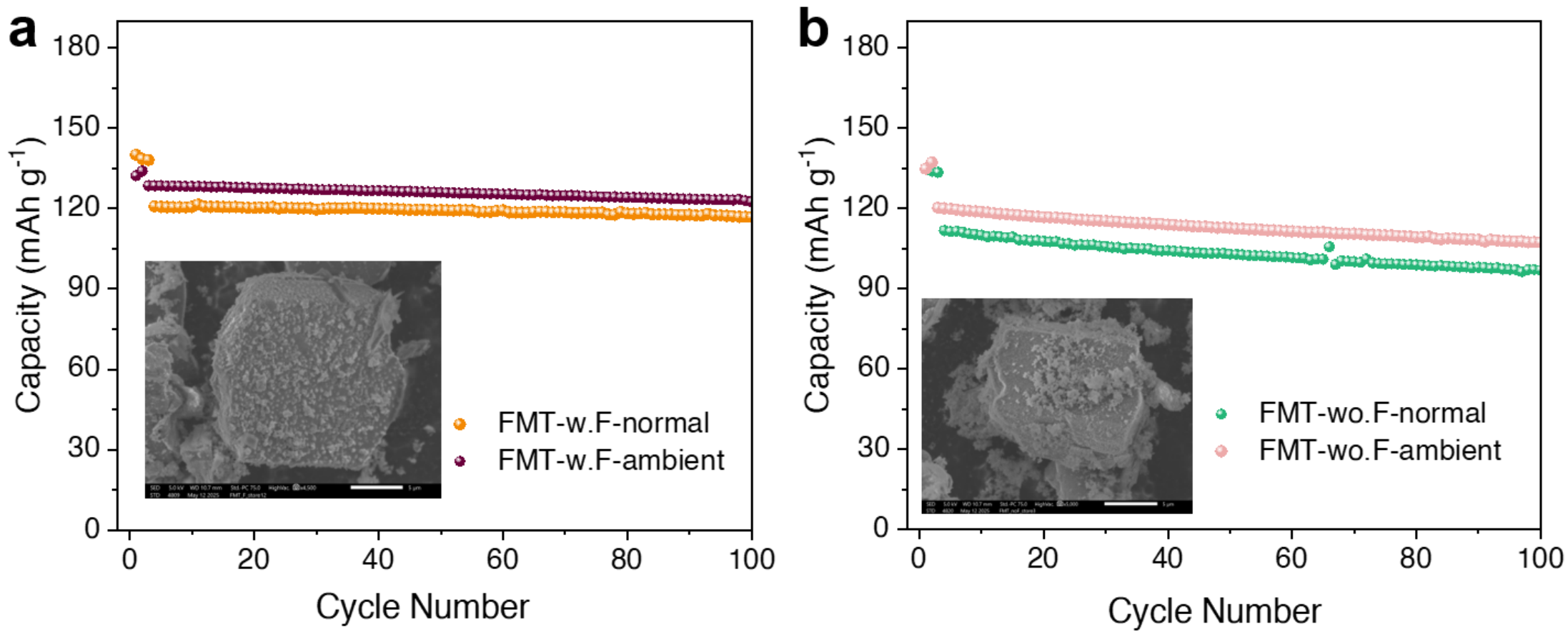

Figure S15. Parallel evaluation of the ambient-storage stability of FMT-w.F and FMT-wo.F. (a) Cycling performance of pristine and air-exposed FMT-w.F after storage under ambient conditions for 14 days, together with the corresponding SEM image of the exposed particle. (b) Cycling performance of pristine and air-exposed FMT-wo.F after the same 14-day ambient exposure (cycled at 0.5 C within 1.5-4.0 V, with the initial two formation cycles at 0.1 C), together with the corresponding SEM image. All cells were tested at 25°C, with the electrolyte composed of 1M $NaPF_6$ in diglyme.

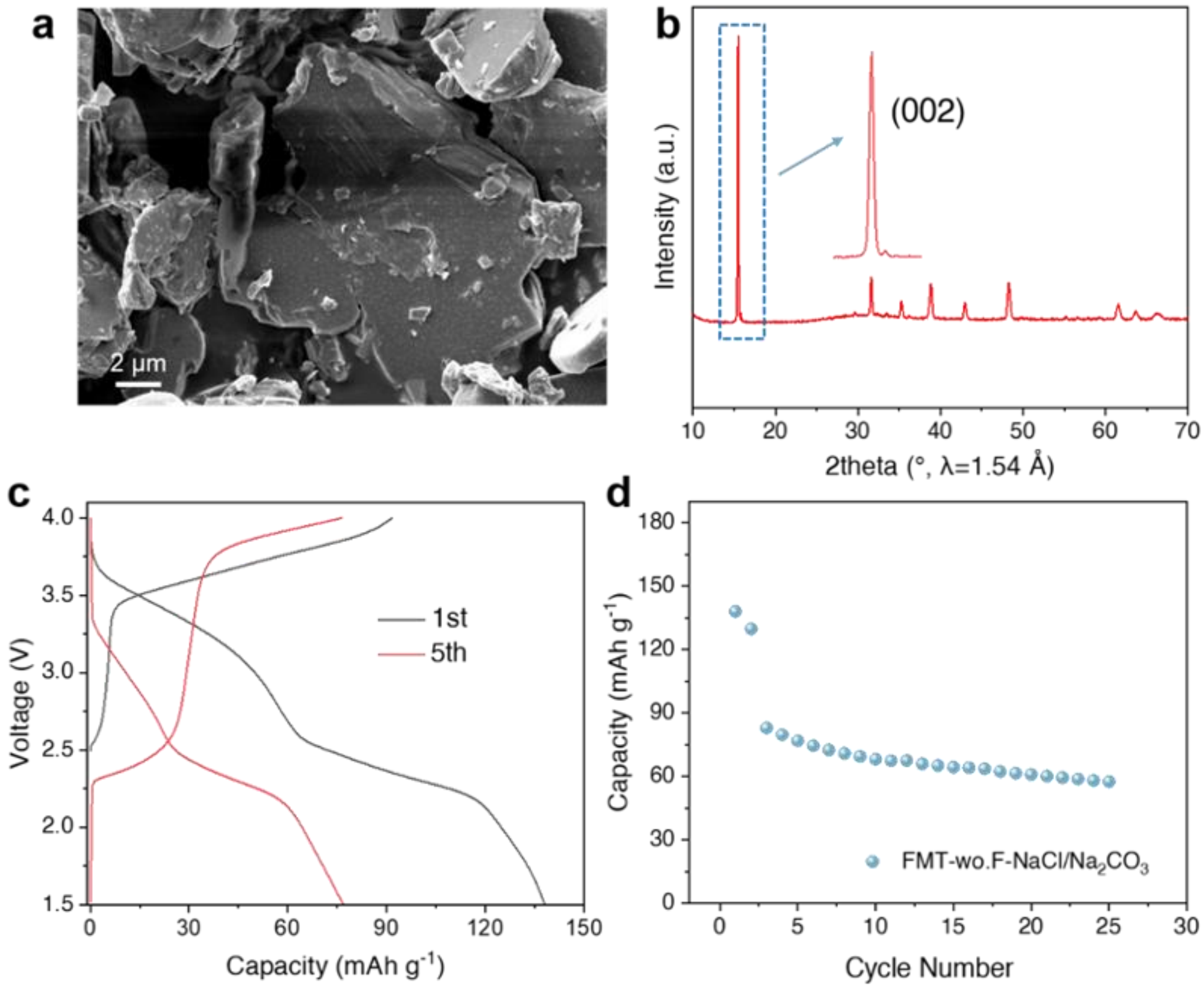

Figure S16. Morphological, structural, and electrochemical characterization of the F-free NaCl/Na_2CO_3 molten-salt control. (a) SEM image showing that the NaCl/Na_2CO_3 molten-salt medium also promotes the formation of relatively large particles. (b) XRD pattern of the obtained product. The highlighted region and enlarged profile show additional reflections, indicating that the F-free molten-salt treatment does not produce a phase-pure P2 structure. (c) Charge–discharge voltage profile of 5th cycle and (d) cycling performance for the F-free molten-salt control under the same electrochemical testing conditions used for FMT-w.F and FMT-wo.F (0.5 C, 1.5-4.0 V, 25 °C). Although the F-free molten-salt medium facilitates particle growth, the resulting sample exhibits inferior phase purity and electrochemical performance, demonstrating that molten-salt fluxing alone cannot account for the structural and electrochemical advantages of FMT-w.F.

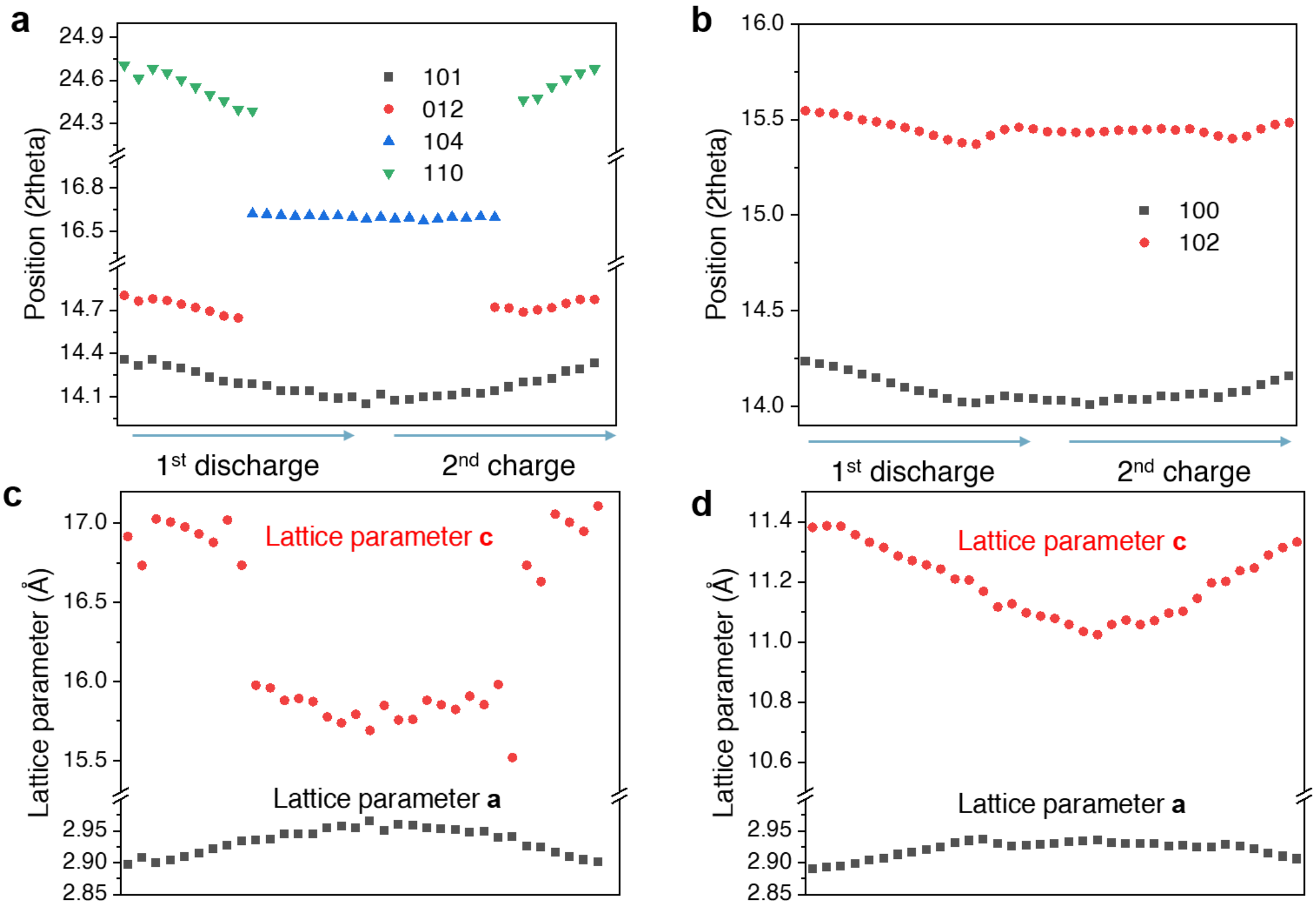

Figure S17. Quantitative analysis of the in-situ XRD evolution of FMT-wo.F and FMT-w.F during the 1st discharge–2nd charge process. (a,b) Evolution of the refined lattice parameters of FMT-wo.F and FMT-w.F, respectively, as a function of the electrochemical state. (c,d) Corresponding evolution of the lattice parameter c and a. The arrows indicate the electrochemical direction. Because of the relatively low signal-to-noise ratio, peak overlap, and the disappearance of several reflections at particular states of charge, the lattice parameters were estimated using only the reliably resolved reflections available at each state. For FMT-wo.F, the analysis was mainly based on the (101), (012), (104), and (110) reflections when observable. The results should therefore be regarded as semi-quantitative and are used primarily to compare the trends and reversibility of structural evolution between the two samples rather than to provide high-precision absolute lattice parameters or phase fractions.

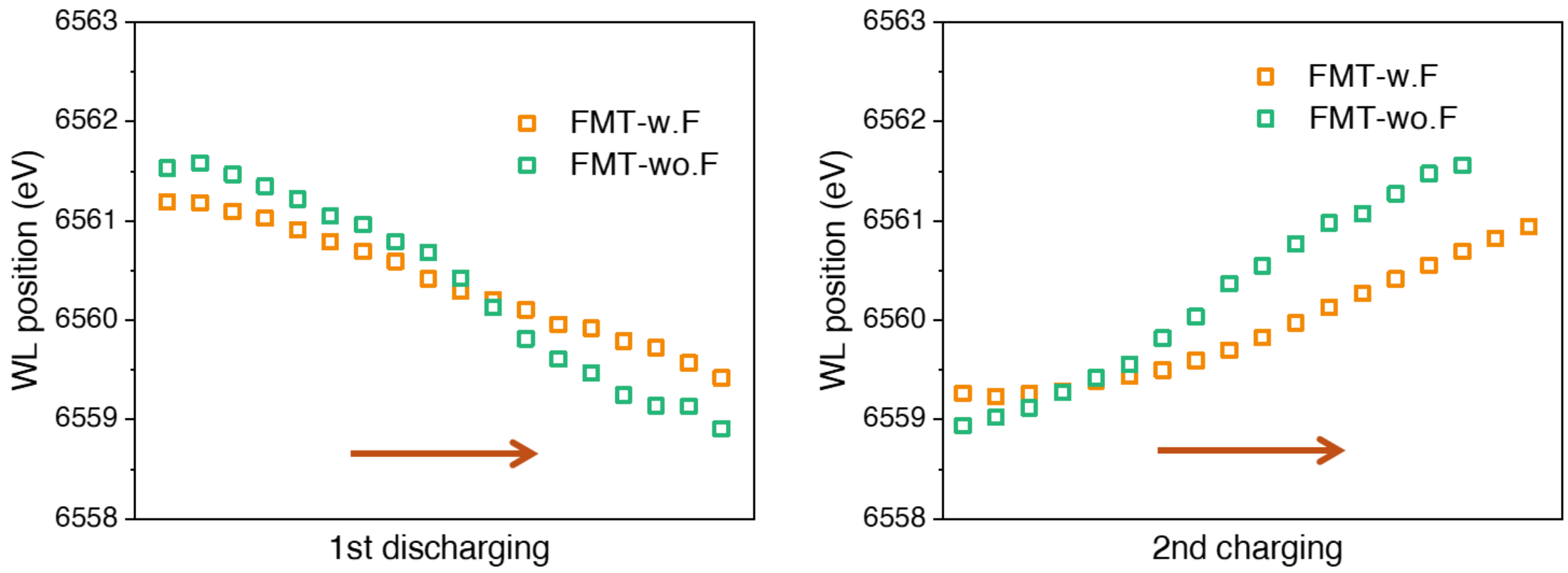

Figure S18. Mn K-edge XANES white-line position variation during the first discharging and second charging processes

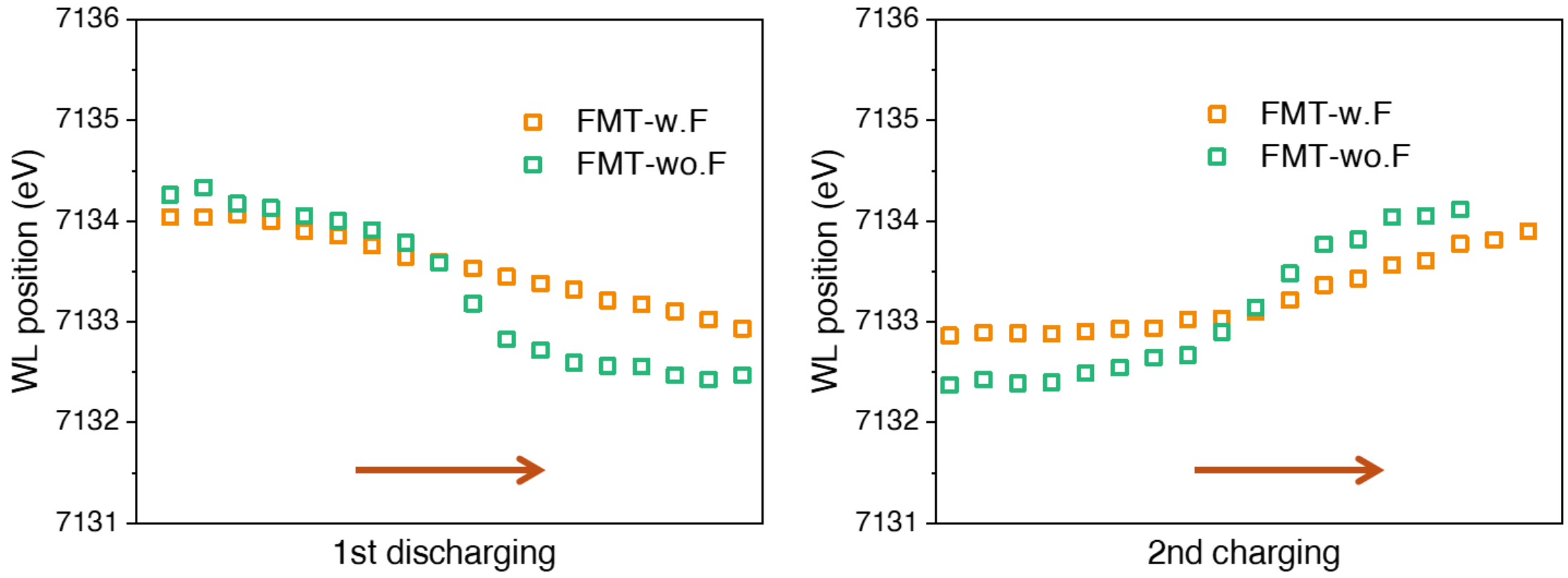

Figure S19. Fe K-edge XANES white-line position variation during the first discharging and second charging processes

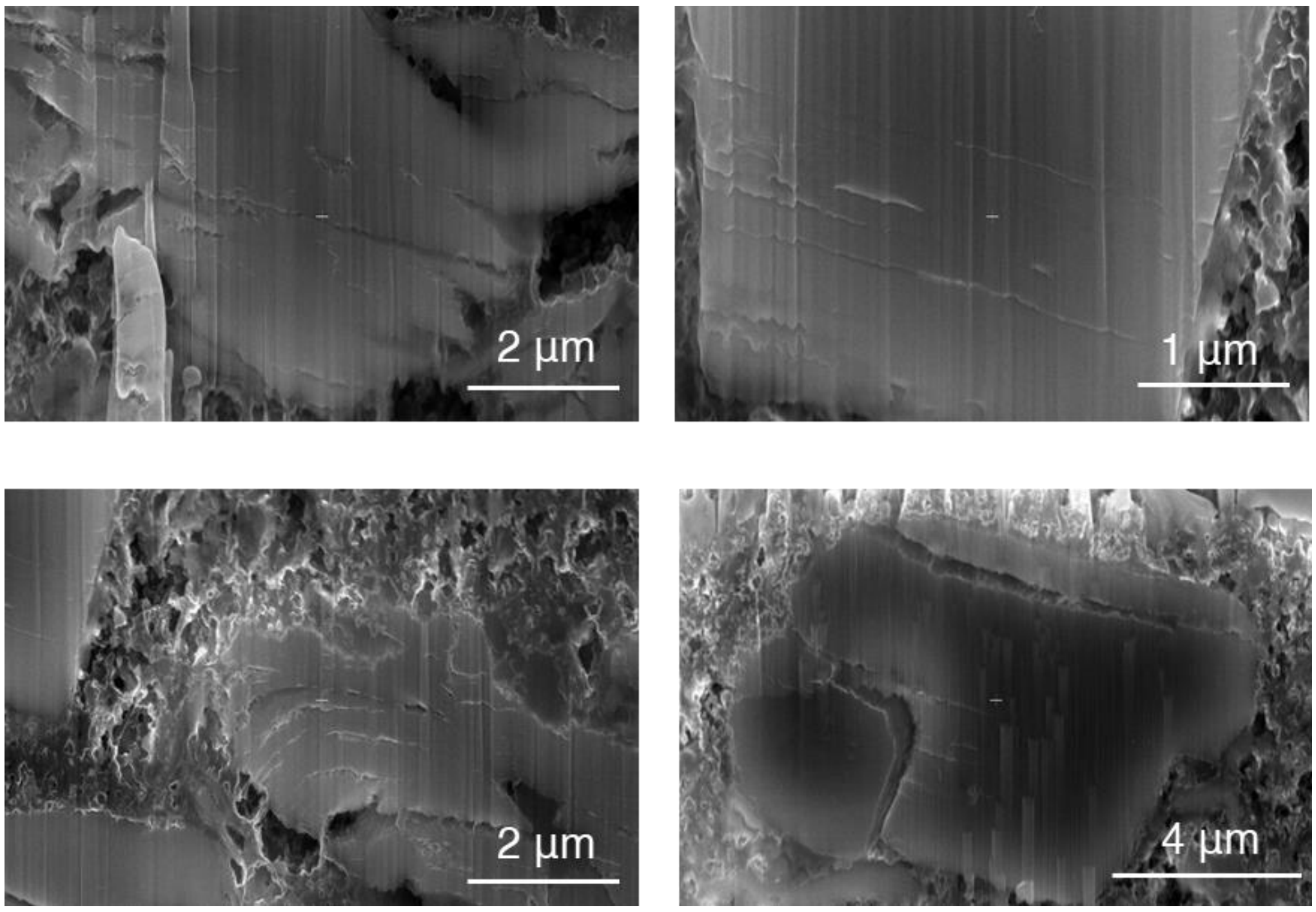

Figure S20. Magnified SEM images showing severe intraparticle damage in cycled FMT-wo.F particles from Fig. 5a, including localized deformation and internal cracking, with several particles displaying band-like or kink-like defect features.

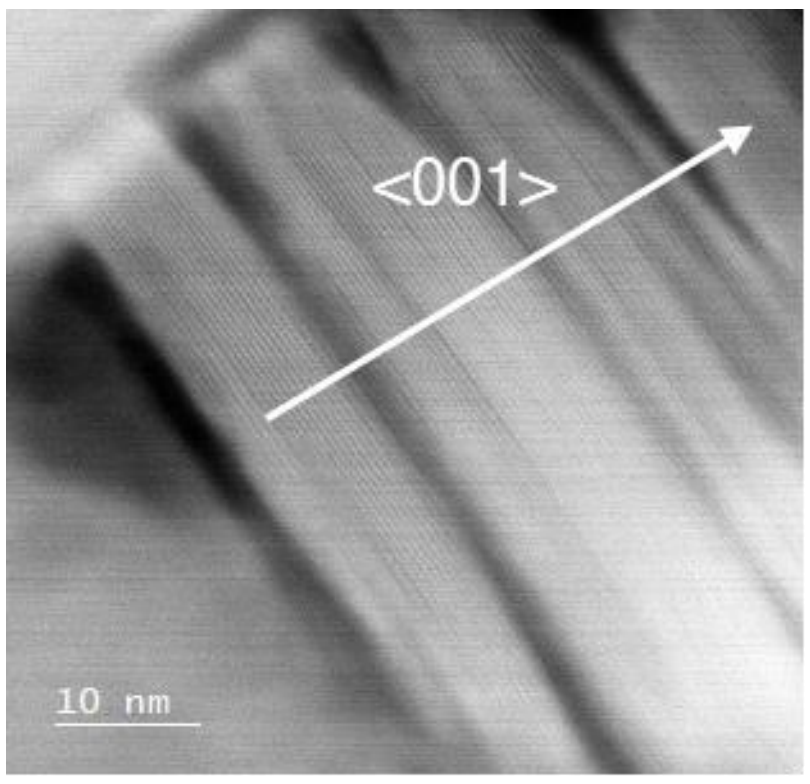

Figure S21. HAADF-STEM image of cycled FMT-wo.F. There are severe intragranular cracks generation.

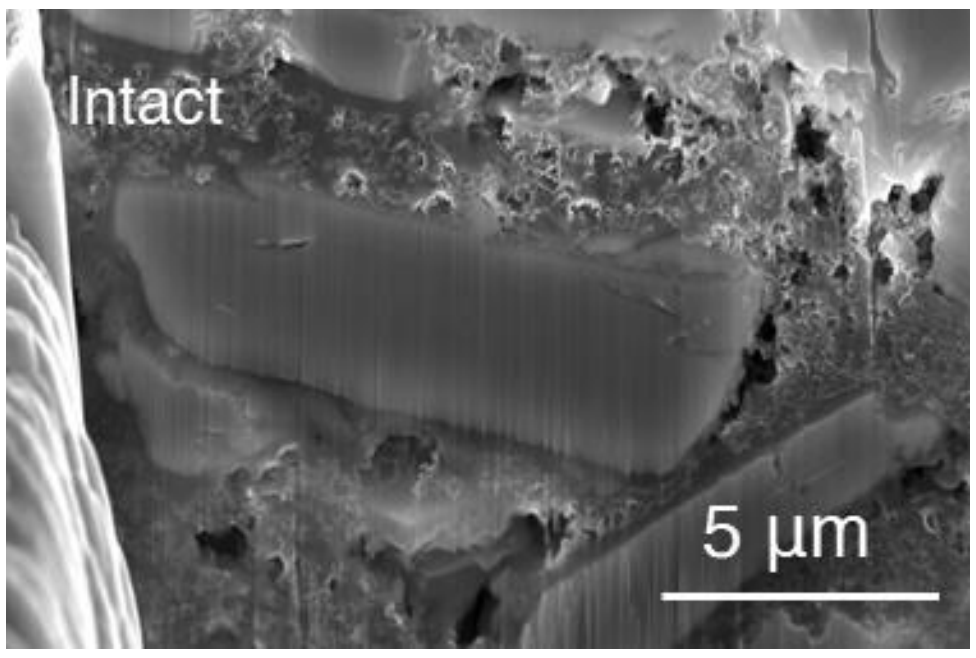

Figure S22. Enlarged view of SEM image for cycled FMT-w.F from Fig. 5d. The particle keeps intact and no visible cracks are generated inside the particle after cycling.

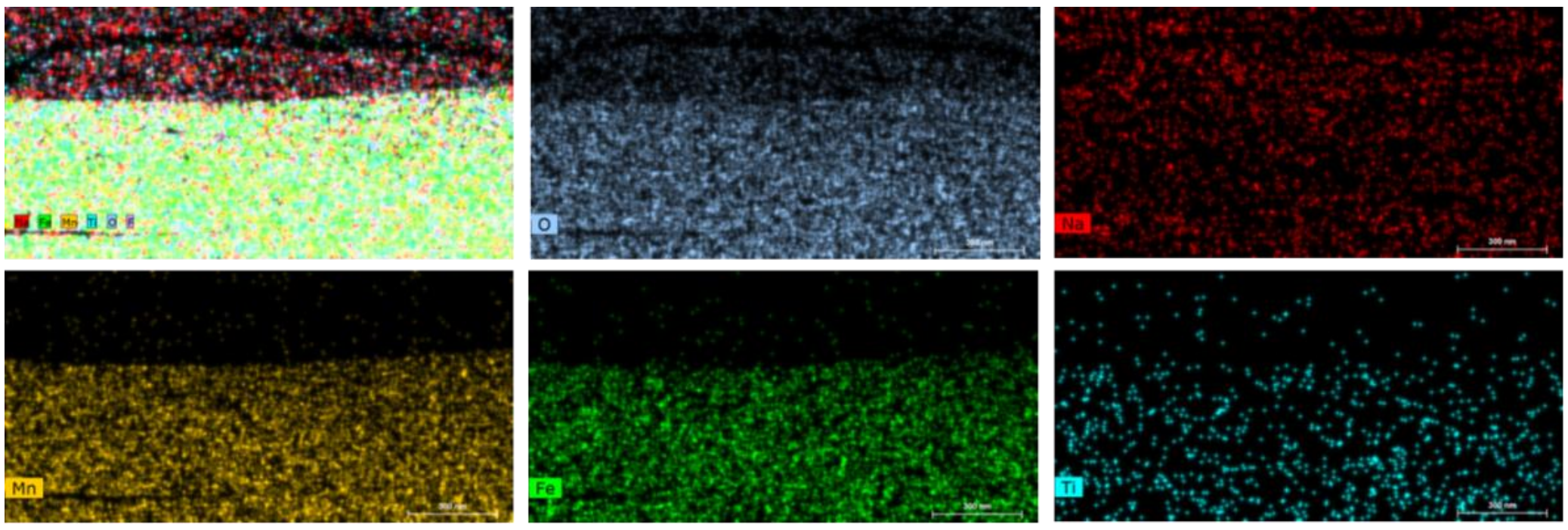

Figure S23. EDS-mapping for cycled FMT-w.F, with F signal showing in Fig. 5e. The element remains homogenous after cycling.

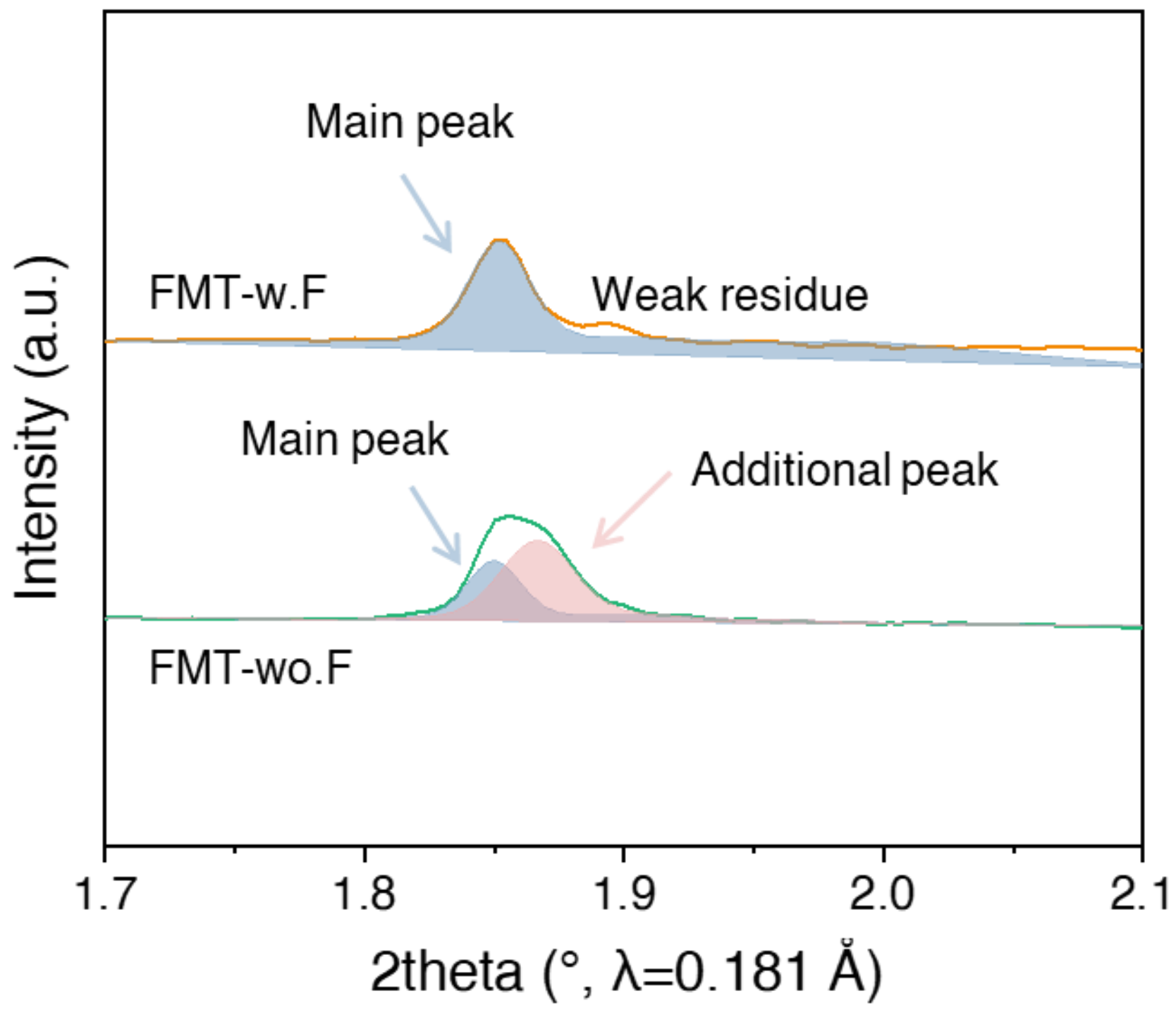

Figure S24. Synchrotron-based XRD patterns of cycled FMT-wo.F and FMT-w.F after 100 cycles in the discharged state. Peak deconvolution of the low-angle peak suggests that FMT-wo.F develops an additional phase, consistent with more heterogeneous and less reversible structural evolution during cycling. In contrast, FMT-w.F is dominated by a single main peak with much weaker residual splitting, suggesting more complete structural recovery and reduced retention of structurally distinct layered configurations after cycling.

Table S1. Elemental composition of FMT-wo.F and FMT-w.F measured by ICP-MS. The fluorine content was estimated by charge balance using the measured Na, Fe, Mn, and Ti compositions together with the average Mn valence derived from the XANES analysis.

	Na	Fe	Mn	Ti
FMT-wo.F	0.769*	0.254	0.661	0.084
FMT-w.F	0.593	0.259	0.664	0.077

* Na content is higher than the actual content due to incomplete sodiation during synthesis, which leaves residual sodium salts on the surface.

Table S2. Formation energy in P2 phase $Na_{0.59}Mn_{0.625}Fe_{0.25}Ti_{0.125}O_2F_{0.03}$.

	E(FMT+F)	E(FMT)	E(O_2)	E(F_2)	E_formation
1-POSCAR	-771.6395	-773.6984	-8.4809	-1.8483	-1.2574
2-POSCAR	-772.6305	-773.4609	-8.4809	-1.8483	-2.4859

Table S3. Comparison of compositions or valence-related information inferred from different experimental and computational methods for FMT-wo.F and FMT-w.F. Because these methods probe different length scales and rely on different assumptions, the exact chemical stoichiometry cannot be uniquely assigned from any single method.

Method	Direct observable	Probing scale / sensitivity	Main assumption used for interpretation	What it tells us for FMT-w.F vs. FMT-wo.F	Limitation
ICP-MS	Bulk Na, Fe, Mn, Ti elemental ratios	Bulk average	Mn valence taken from XAS; Fe and Ti treated as nominal Fe^{3+} and Ti^{4+}; F inferred from charge neutrality.	Suggests non-negligible F incorporation in FMT-w.F and different bulk Na content between the two samples	F is not directly measured, inferred composition depends on charge-balance assumptions
Hard XAS, Mn K-edge	Edge position and spectral shape	Bulk average, valence-sensitive	Comparison with reference spectra or relative edge shifts	Indicates slightly lower average Mn valence, or more Mn^{3+}-like character, in FMT-w.F	Semi-quantitative, influenced by covalency and local coordination
Soft XAS, Mn L-edge	Surface-sensitive multiplet features	Near-surface region	Spectral line shape reflects relative Mn^{3+}/Mn^{4+}-like contribution	Suggests a higher fraction of Mn^{3+}-like species near the surface of FMT-w.F	Surface-sensitive only, not directly representative of bulk composition
Soft XAS, O K-edge	Pre-edge / hybridization-related features	Near-surface region	Changes in spectral features reflect TM–O hybridization and local anion chemistry	Indicates modified TM–O electronic interactions in FMT-w.F, consistent with F-induced local chemical regulation	Does not provide a direct stoichiometric F content

TXM-XANES	Spatially resolved XANES contrast or fitted valence distribution	Particle scale, spatially resolved	Reference-based fitting or relative spectral comparison	Shows improved spatial electronic uniformity in FMT-w.F	Semi-quantitative, depends on fitting model and reference selection
DFT	Magnetic moment, Bader charge, etc	Idealized model system	Fixed Na content and chosen F configuration for controlled comparison	Supports slightly increased Mn^{3+}-like character and local charge redistribution upon F incorporation	Model composition is simplified and does not exactly reproduce experimental stoichiometry

Table S4 Electrochemical performance comparison of FMT-w.F and representative Ni/Co-free P2-type sodium layered oxide cathodes reported in the literature. The listed parameters include composition, voltage window, current density or C-rate, specific capacity, capacity retention, cycle number, and reference. Performance values are taken directly from the original publications, and differences in testing conditions should be considered when making comparisons.

Composition	Capacity	Retention	Voltage range	C-rate	Ref.
P2-$Na_{2/3}Fe_{1/2}Mn_{1/2}O_2$	126 mAh g^{-1}	75% after 30 cycles	1.5–4.0 V	0.1 C (26 mA g^{-1})	*ACS Appl. Mater. Interfaces* 2016, 8 (4): 2857–2865.
P2-$Na_{2/3}Fe_{1/2}Mn_{1/2}O_2$	145 mAh g^{-1}	94% after 30 cycles	2.0–4.2 V	0.1 C	*RSC Adv.* 2014, 4 , 22798–22802
P2-$Na_{0.67}Fe_{0.5}Mn_{0.5}O_2$	120 mAh g^{-1}	81.25 % after 150 cycles	1.5–4.0 V	1 C (260 mA g^{-1})	*J. Colloid Interface Sci.* 2025, 685, 87–96
Ti-substituted P2-$Na_{0.67}Fe_{0.5}Mn_{0.5}O_2$	~110 mAh g^{-1}	64% after 45 cycles	1.5–4.2 V	0.5 C (130 mA g^{-1})	*ACS Omega.* 2018, 3(1):361–368
P2-$Na_{2/3}Mn_{0.8}Fe_{0.1}Ti_{0.1}O_2$	99 mAh g^{-1}	87.7% after 300 cycles	2.0–4.0 V	1 C (245 mA g^{-1})	*Chem. Mater.* 2016, 28 (1): 106–116
P2-$Na_{2/3}Mn_{0.8}Fe_{0.1}Ti_{0.1}O_2$	74 mAh g^{-1}	77% after 7 cycles	1.5–4.2 V	2 C (263 mA g^{-1})	*J. Mater. Chem. A* 2019, 7(19): 12115–12125
P2-$Na_{2/3}Mn_{7/12}Fe_{1/3}Ti_{1/12}O_2$	170 mAh g^{-1}	80% after 50 cycles	1.5−4.3 V	0.1 C (17 mA g^{-1})	*Chem. Mater.* 2024, 36, 4107−4120
Al-substituted $Na_{0.67}Fe_{0.5}Mn_{0.5}O_2$	~160 mAh g^{-1}	76% after 50 cycles	1.5–4.2 V	0.1 C (20 mA g^{-1})	*Inorg. Chem.* 2018, 57 (9): 5249–5257
Na_2SiO_3-coated/Si-doped P2-$Na_{0.67}Fe_{0.5}Mn_{0.5}O_2$	~168 mAh g^{-1}	63% after 100 cycles	1.5–4.2 V	0.1 C (26 mA g^{-1})	*Electrochim. Acta* 2021, 384, 138362
K-doped P2-$Na_{0.67}Fe_{0.5}Mn_{0.5}O_2$	109 mAh g^{-1}	70% after 100 cycles	1.5–4.2 V	1 C	*J. Alloys Compd.* 2023, 947,169482
Sn Doping and LiF Coating P2-	184 mAh g^{-1}	68% after 100 cycles	1.5–4.2 V	2 C (260 mA	*Adv. Funct. Mater.* 2026, 36, e75689

$Na_{0.67}Fe_{0.5}Mn_{0.5}O_2$				g^{-1})	
P2-$Na_{0.67}Fe_{0.5}Mn_{0.5}O_2$	126 mAh g^{-1}	91% after 100 cycles	1.8–4.4V	1 C (260 mA g^{-1})	*Chem. Eng. J.* 2025, 524, 169178
P2-$Na_{2/3}Mn_{0.8}Fe_{0.1}Mg_{0.1}O_2$	156 mAh g^{-1}	90% after 50 cycles	1.5–4.2 V	1 C (240 mA g^{-1})	*Ionics* 2020, 26:735–743
P2-$Na_{0.7}Cu_{0.15}Fe_{0.3}Mn_{0.55}O_2$	97.8 mAh g^{-1}	82% after 80 cycles	2.5–4.2 V	0.1 C	*J. Mater. Chem. A* 2018, 6(42), 20795–20803
P2-$Na_{0.67}Mn_{0.6}Cu_{0.3}Mg_{0.1}O_2$	121 mAh g^{-1}	86 % after 100 cycles	2.0–4.5 V	0.2 C (34 mA g^{-1})	*Chem. Eng. J.* 2023, 452, 138912
P2-$Na_{0.67}Mn_{0.62}Al_{0.05}Fe_{0.31}La_{0.02}O_2$	128.7 mAh g^{-1}	85 % after 100 cycles	1.5–4.3 V	1 C (175 mA g^{-1})	*J. Energy Storage* 2026, 160, 121971
O3/P2-$Na_{0.75}Mn_{0.4}Fe_{0.4}Ti_{0.1}Al_{0.1}O_2$	148 mAh g^{-1}	92 % after 50 cycles	2.2–4.2 V	25 mA g^{-1}	*Commun. Mater.* 2023, 4, 6
O3/P2-$Na_{0.75}Mn_{0.35}Fe_{0.35}Ti_{0.1}Al_{0.1}Cu_{0.1}O_2$	140 mAh g^{-1}	80 % after 70 cycles	2.2–4.2 V	25 mA g^{-1}	*Batter. Supercaps* 2023, 6, e202300089
$Na_{0.593}Fe_{0.259}Mn_{0.664}Ti_{0.077}O_2F_{0.13}$	172 mAh g^{-1}<br>140 mAh g^{-1}<br>89 mAh g^{-1}	88 % after 50 cycles<br>87 % after 240 cycles<br>82 % after 500 cycles	1.5–4.3 V<br>1.5–4.0 V<br>2.0–4.0 V	0.5 C (65 mA g^{-1})	This work

References

1. Xiao, X.; Xu, Z.; Lin, F.; Lee, W. K. TXM-Sandbox, an open-source software for transmission X-ray microscopy data analysis. *J. Synchrotron Radiat.* **2022**, *29*, 266-275.
2. Grindy, S.; Meredig, B.; Kirklin, S.; Saal, J. E.; Wolverton C. Approaching chemical accuracy with density functional calculations: Diatomic energy corrections. *Phys. Rev. B* **2013**, *87*, 075150.
3. Bianchini, M.; Wang, J.; Clément, R. J.; Ouyang, B.; Xiao, P.; Kitchaev, D.; Shi, T.; Zhang, Y.; Wang, Y.; Kim, H.; Zhang, M.; Bai, J.; Wang, F.; Sun, W.; Ceder, G. The interplay between thermodynamics and kinetics in the solid-state synthesis of layered oxides. *Nat. Mater.* **2020**, *19*, 1088-1095.
4. FactSage. Na_2CO_3-NaF Phase Diagram Database. https://www.crct.polymtl.ca/fact/phase_diagram.php?file=Na2CO3-NaF.jpg&dir=FTsalt (accessed August 2, 2026).
5. Kang, K.; Zhang, H.; Kim, J. H.; Byun, W. J.; Lee, J. S. An In Situ Fluorine and Ex Situ Titanium Two-Step Co-Doping Strategy for Efficient Solar Water Splitting by Hematite Photoanodes. *Nanoscale Adv.* **2022**, *4*, 1659–1667.
6. Huang, Y.; Ding, R.; Ying, D.; Shi, W.; Huang, Y.; Tan, C.; Sun, X.; Gao, P.; Liu, E. Engineering Doping–Vacancy Double Defects and Insights into the Conversion Mechanisms of an Mn–O–F Ultrafine Nanowire Anode for Enhanced Li/Na-Ion Storage and Hybrid Capacitors. *Nanoscale Adv.* **2019**, *1*, 4669–4678.
7. Li, K.; Zhang, J.; Lin, D.; Wang, D.-W.; Li, B.; Lv, W.; Sun, S.; He, Y.-B.; Kang, F.; Yang, Q.-H.; Zhou, L.; Zhang, T.-Y. Evolution of the Electrochemical Interface in Sodium Ion Batteries with Ether Electrolytes. *Nat. Commun.* **2019**, *10*, 725.
